# Unveiling the crosstalk between unfolded protein response and apoptosis in triclosan induced hepatotoxicity in *Labeo rohita*

**DOI:** 10.1038/s41598-025-93997-0

**Published:** 2025-05-16

**Authors:** Abha Trivedi, Vaishnavi Saxena, Mahdi Banaee, Jumman Bakhasha, Neeti Arya, Kamlesh K. Yadav, Maria Giovanna Rizzo, Caterina Faggio

**Affiliations:** 1 Toxicogenomics Laboratory, Department of Animal Science, M.J.P. Rohilkhand University, Bareilly, 243006 India; 2 Department of Zoology, Government Degree College, Bakkha Kheda, Unnao, 209801 India; 3 Department of Environmental Sciences, Faculty of Natural Resources, Behbahan Khatam Alanbia University of Technology, Behbahan, Iran; 4 Department of Chemical, Biological, Pharmaceutical and Environmental Sciences, University of Messina, Messina, Italy; 5 Department of Eco-Sustainable Marine Biotechnology, Stazione Zoologica Anton Dohrn, Naples, Italy

**Keywords:** Triclosan, Fish, Endoplasmic reticulum stress, PERK pathway, Unfolded protein response, Zoology, Environmental sciences

## Abstract

Triclosan (TCS), a widely used antimicrobial, has emerged as a concerning aquatic pollutant, especially post-COVID-19 due to increased disinfectant use. The study aimed to investigate the toxic impacts of TCS on *Labeo rohita*, revealing a 96-h LC_50_ of 0.742 mg/L. Well-acclimatized fish were categorised into three groups: one control and two treatment (T1 and T2) that were exposed to 1/10th and 1/5th of 96-h LC_50_ i.e., 0.0742 mg/L and 0.148 mg/L, respectively for 6 weeks under semi-static condition. Significantly (*p* < 0.05) elevated extents of reactive oxygen species (ROS) in the liver indicated enhanced oxidative stress. The activities of enzymatic antioxidants viz. superoxide dismutase (SOD) and catalase (CAT) were significantly (*p* < 0.05) increased while the reduced glutathione (GSH) levels were significantly (*p* < 0.05) decreased in a dose- and duration-dependent manner. A significant (*p* < 0.05) increase was observed in the lipid peroxidation (LPO) rate, which coincided with disruptions in the histological structure of the liver. Significant (*p* < 0.05) induction in Micronuclei frequency validated genomic instability. Furthermore, Endoplasmic Reticulum (ER) stress was marked by the increased intracellular calcium levels and elevated expression of *grp78*,* chop*,* atf4*,* perk*,* eIF2α* and *gadd34* in TCS-exposed groups. Moreover, the consequent activation of *bax*,* caspase-3*,* caspase-9*,* apaf-1* and the regulation of *bcl-2* due to ER-stress, ultimately resulted in apoptosis of hepatocytes. Pearson correlation matrix and Principal Component Analysis (PCA) further underscored key interactions among parameters. Our findings demonstrate that the TCS, at its sub-lethal concentrations, is causing severe anomalies and even cell-death via triggering ER-stress-mediated apoptotic pathway in the fish liver.

**Supplementary Information:**

The online version contains supplementary material available at 10.1038/s41598-025-93997-0.

## Introduction

Triclosan (TCS) is a synthetic and lipophilic compound with the IUPAC name is 2,4,4′-trichloro-2′-hydroxydiphenyl ether or 5-chloro-2-(2,4-dichlorophenoxy) phenol. It is widely used globally as an ingredient in disinfectants, soaps, detergents, toothpaste, mouthwash, fabrics, deodorant, shampoo and plastic additives, in addition to numberless other personal care, veterinary, pharmaceutical, industrial and household products, due to its antimicrobial properties^1^. Moreover, the use of TCS as a household sanitizing agent, in kitchenware, textiles and plastic toys has also been reported^2^.

Due to misuse, overuse, slow absorption, high water solubility and resistance to degradation, anti-microbial agents are often pervasive in the environment^3^. The widespread use of TCS in various products creates multiple pathways for the compound to enter the environment, particularly through wastewater. Studies have shown that since the COVID-19 pandemic, the heightened use and constant release of TCS from soaps and sanitizers have increased considerably^4^. While TCS plays an important role in infection prevention and has been effective in controlling microbial growth in medical and consumer products, its accumulation in the environment has raised concerns due to potential risks, including antimicrobial resistance, endocrine disruption, bioaccumulation in aquatic ecosystems, and toxicity to non-target organisms, including humans^5^. Furthermore, developing alternatives that maintain antimicrobial effectiveness while minimizing ecological impact remains a significant challenge. These issues highlight the need for a careful evaluation of TCS’s benefits and potential hazards to both human health and the environment^6^.

Approximately 96% of TCS from consumer products is discharged into residential drains, which subsequently enter the wastewater treatment plants (WWTP)^7,8^. Inappropriate wastewater treatment and continuous sewage emanations into river surface water ultimately contaminate aquatic ecosystems^4^. Due to COVID-19 measures, the worldwide river contamination with TCS was reported to be enhanced by 33%^9^. Its presence has been detected in various biological fluids, including urine, breast milk and blood plasma, as well as tissues like the liver, placenta, adipose and brain in various quantities^10,11^.

As an antibacterial agent, TCS targets multiple sites within cells and the cytoplasm, potentially interfering with gene transcription and disrupting signaling pathways^12^. The Endoplasmic Reticulum (ER), a crucial intracellular organelle, plays a vital role in protein synthesis, folding, modification, lipid metabolism, and calcium regulation^13,14^. However, various environmental factors, physiological issues, infections, DNA damage, and xenobiotics can disrupt ER homeostasis, leading to ER stress^15,16^. ER stress is characterized by the accumulation of misfolded and unfolded proteins in the ER lumen^17^. In response, cells activate the unfolded protein response (UPR) to alleviate this protein overload and sustain cellular function. The UPR involves three primary signaling pathways: inositol-requiring enzyme 1α (IRE1α), activating transcription factor 6α (ATF6α), and protein kinase-like endoplasmic reticulum kinase (PERK)^18^. Although the UPR is essential for managing protein accumulation, prolonged activation of this response can ultimately lead to cell death^19^.

Due to its lipophilic properties, TCS has the capacity to bioaccumulate in the tissues of various organisms and transfer across the food web, leading to potential long-term toxicity^11^. Studies have reported bioaccumulation of TCS in different species, including fish, invertebrates, and mammals, demonstrating its widespread persistence^20^. Even at low concentrations, TCS poses risks to human health by disrupting hormone regulation and potentially causing developmental and immune issues. Additionally, it threatens the environment by inducing oxidative stress, causing transcriptional alterations in aquatic organisms, and contributing to antimicrobial resistance^21–23^. Its accumulation in food fish (*Cyprinus carpio*), aquarium fish (*Carassius auratus*), and predatory species (*Tigriopus japonicus*), disrupting essential physiological functions, endangering both freshwater ecosystems (e.g., *Danio rerio*) and marine life (e.g., *Diplodus sargus*), eventually posing serious health risks to humans through bio-magnification^24,25^. Elevated TCS levels can increase mortality rates among fish, leading to population declines and adversely impacting overall aquatic biodiversity^26,27^. This disruption threatens the survival of various species and jeopardizes the health of ecosystems that rely on diverse aquatic life.

Therefore, acknowledging the importance of TCS toxicity in aquaculture, there has been a remarkable increase in research investigating its effects on aquatic organisms. Using *Labeo rohita* as the model organism, this study provides valuable insights into the toxicological effects of TCS in aquatic species. *L. rohita*, a widely cultivated freshwater fish, is ecologically and economically significant, making it an ideal species for assessing environmental contaminants. Its high sensitivity to waterborne pollutants, well-studied physiology, and relevance in aquaculture and food security further enhance its suitability for toxicological research. Although many studies have explored the impact of TCS on aquatic life, gaps persist in our understanding of TCS-induced cell death mediated by endoplasmic reticulum (ER) stress in fish^28–33^. The present study specifically aims to clarify the mechanisms behind TCS-induced hepatic apoptosis, focusing particularly on the role of ER stress via PERK pathway. The results could have important implications for environmental health and inform regulatory policies regarding the use and disposal of antimicrobial agents like TCS.

## Materials and methods

### Test chemical

Triclosan (CAS number: − 5 and purity ≥ 97%) was bought from Sigma Aldrich. Its stock solution was made in Di-methyl sulphoxide (DMSO) at a concentration of 10 mg/ml and stored at room temperature (25 ± 1 °C).

### Chemicals used

Ethylenediaminetetraacetic acid, dimethyl sulfoxide, potassium per magnet, trichloroacetic acid, hydrochloric acid, ethanol, trizol, chloroform, isopropanol, thiobarbituric acid, DEPC water, tris base, phenylmethylsulfonyl fluoride, Dithiothreitol, dithiobis (2-nitrobenzoic acid), phenazine methosulfate, nitroblue tetrazolium, Nicotinamide adenine dinucleotide reduced disodium salt hydrate, hydrogen peroxide, methanol, Giemsa stain, may-Grunwald’s, dichlorodihydrofluorescein diacetate, acetic acid, ethidium bromide, and dibutyl phthalate polystyrene xylene.

### Sample size calculation

The sample size was calculated using GPower 3.1 software^34^, based on ANOVA for repeated measures with between-subject effects with a 5% significance level, 90% power, and a medium effect size (0.5), the required sample size was 90 fish.

### Test model and acclimatization

*L. rohita* (18.23 ± 2.12 g and 14.57 ± 1.35 cm long) were collected with the help of local fishermen and imported to the laboratory. Procured fish were carefully washed with 0.05% potassium permanganate (KMnO_4_) to abolish possible cutaneous infections^35^ and acclimatized in 120 L glass aquaria for 15d, then, transferred to 100 L well-aerated glass aquaria (100 × 40 × 40cm^3^), containing 15d aged 40 L tap water. Fish were fed twice daily with commercial aquarium food pellets (Perfect Companion Group Company Limited, Thailand) at the rate of 3% of the body weight per day^36^. Feeding was halted one day before the start of the experiment to ensure uniform conditions^37^. The aquaria were cleaned, and the water was renewed regularly to maintain optimal water quality^38^. Residual food particles and faecal matter were also removed daily^39^.

### Acute exposure

96-h LC_50_was recorded under acute exposure as per the guidelines of the American Public Health Association (APHA)^40^and Organization for Economic Co-operation and Development guidelines for fish acute bioassays (OECD203, 92/69/EC, method C1)^37^. Sixty equal-sized fish were placed in six different concentrations of TCS (10 fish in each) i.e., 0.2, 0.4, 0.6, 0.8, 1.0, and 1.2 mg/L for 96-h for the major toxicity range. 100% mortality in the concentrations of 0.8, 1.0, and 1.2 mg/L while no mortality was reported in the 0.2, 0.4, and 0.6 mg/L in 96-h. To obtain a decisive concentration, another 60 fish were randomly loaded in six aquaria (10 fish in each) that had TCS concentrations below 0.8 mg/L i.e., 0.68, 0.7, 0.72, 0.74, 0.76, and 0.78 mg/L for 96-h. The mortality rate was reported every 24 h and dead fish were removed by hand net. The Probit analysis method^41^ was followed to ascertain the 96-h LC_50_ of TCS.

### Chronic exposure

Ninety well-habituated fish were segregated into three groups in triplicates that contained 10 fish each. One group was kept as control and other two groups, i.e., T1 and T2 were subjected to 0.0742 mg/L (1/10th 96-h LC_50_) and 0.148 mg/L (1/5th 96-h LC_50_) TCS-exposure, respectively, for 2, 4 and 6 weeks, to ensure sub-lethal yet biologically relevant exposure, a standard approach in toxicological research. These concentrations allow us to examine chronic toxicity and mechanistic pathways without causing acute mortality, making the findings more applicable to real-world environmental scenarios. No mortality was observed during the whole experiment in all groups. During each sampling (at 2, 4, and 6 weeks), three fish from each replicate were anesthetized with tricaine methane sulfonate (MS-222; 0.3 g/L, Sigma Aldrich E10521)^42^. Blood was collected from the caudal vessel using a heparinized syringe and stored in EDTA-coated vials (1.8 mg/mL) for micronucleus assay. The liver was anatomized for further assessments viz. estimation of ROS, oxidative stress, histopathological investigations and transcriptional profiling. Sampling was conducted in accordance with OECD guidelines^43^.

### Ethics

All fish experiments were approved and conducted in accordance with the Institutional Animal Ethics Committee (IAEC) Regd. No. MJPRU/PY/IAEC/22/20 CPCSEA at M. J. P. Rohilkhand University, Bareilly, adhering to the guidelines set by the Committee for Control and Supervision of Experiments on Animals (CPCSEA), Government of India and the study adhered to the ARRIVE guidelines.

### Measurement of ROS in the liver

DCFH-DA dye was used to estimate the ROS levels in all experimental groups. The liver samples were washed with cold PBS (pH 7.4), homogenized and centrifuged at 12,000 g for 30 min. 20 µl supernatant was collected in tubes and incubated at room temperature (25 ± 1 °C) for 5 min. 100 µl PBS and 2 µl working solution of DCFH-DA dye was added to each tube and incubated for 30 min in dark^44^. Afterwards, the intracellular fluorescence was assessed using a fluorescence microscope (Leica DM750, Germany), at excitation and emission wavelengths of 482 nm and 532 nm, respectively with 10/40X magnification. Fluorescent intensity was quantified using Image J software (version 1.50, USA).

### Estimation of oxidative stress-related biomarkers

After each experimental period, the liver was extracted and homogenized using a tissue homogenizer. The obtained homogenates were used to quantify oxidative stress biomarkers viz. SOD, CAT, GSH, and LPO with the help of UV-VIS spectrophotometer (Shimadzu, UV-1900i) and their activities were measured in Units/min/mg of protein^45^. The extents of enzymatic stress biomarkers viz. SOD and CAT were evaluated at the wavelength of 560 nm and 240 nm, as per the procedures of Kakkar^46^and Aebi^47^; respectively while the level of non-enzymatic stress biomarker i.e., GSH was measured at 412 nm applying the method of Moron^48^. LPO activity was quantified at 532 nm using the approach of Ohkawa^49^.

### Micronucleus assay

Blood samples were collected from each group, and a uniform blood smear was prepared on pre-cleaned glass slides following the method described by Schmid^50^. For the evaluation of MN percentage, the slides were examined under a microscope, and 2,000 erythrocytes were counted per slide to ensure accurate identification and quantification of MN^51^.

$$\:\text{M}\text{N}{\%}=\frac{\left(\text{n}\text{u}\text{m}\text{b}\text{e}\text{r}\:\text{o}\text{f}\:\text{c}\text{e}\text{l}\text{l}\text{s}\:\text{c}\text{o}\text{n}\text{t}\text{a}\text{i}\text{n}\text{i}\text{n}\text{g}\:\text{M}\text{N}\right)}{\text{t}\text{o}\text{t}\text{a}\text{l}\:\text{n}\text{u}\text{m}\text{b}\text{e}\text{r}\:\text{o}\text{f}\:\text{c}\text{e}\text{l}\text{l}\text{s}\:\text{c}\text{o}\text{u}\text{n}\text{t}\text{e}\text{d}}\times\:100\:$$

### Estimation of calcium content

The levels of calcium (Ca) in the fish liver were determined using Inductively Coupled Plasma Mass Spectrometry (ICP-MS) Optima 7300DV^52^. The results were expressed as mg/kg tissue.

### Hepato-architectural investigations

The liver was properly dissected from the fish of each group, then washed with distilled water and fixed in 10% neutral buffered formalin (NBF) for 48 h. Subsequently, tissues were dehydrated with a graded series of ethanol, cleared with xylol and inserted in paraffin wax to make blocks. The blocks were kept overnight at room temperature (25 ± 1 °C) and trimmed using Microtome (YSI062 Yorco Precision Rotary Microtome, India) to obtain sections of 3 μm which were further flattened on hot plate, then stained and counterstained with hematoxylin and eosin, respectively. Afterwards, slides were prepared via mounting of processed sections with Dibutylphthalate Polystyrene Xylene (DPX) and photographs were captured utilizing an oil immersion microscope (Zeiss Primostar 1) with 10/40X magnification.

### Isolation of RNA and cDNA synthesis

From the fish liver of all experimental groups, total RNA was obtained and analysed for transcriptional profiling using the TRIzol reagent (Life Technologies, Invitrogen, USA). The synthesis of complementary DNA (cDNA) was carried out employing ABScript II cDNA First strand synthesis kit (cat no. RK20400) according to the manufacturer’s recommendations. Integrated DNA Technologies (https://eu.idtdna.com/site) provided the primer pairs i.e., forward and reverse sequences for quantitative Real-Time Polymerase Chain Reaction (qRT-PCR). The list of primer pair sequences used in our study is given in Supplementary Table-1. SYBR green PCR master mix (KM4100, KAPA Biosystems) was applied to examine the expression patterns of genes with the help of a Real-Time PCR (StepOnePlus^®^, Applied Biosystems). Post-completion of reaction, data was collected and the relative quantification of gene transcripts was assessed by means of the 2^-ΔΔCt^method^53^ where Ct is the threshold cycle number i.e., the number of cycles at which the fluorescent signal became more prominent than the background. Gene expression records were normalized using a housekeeping gene i.e., β-actin. PCR products were also observed on 1% agarose gel aiding horizontal gel electrophoresis (Bio-Rad, USA).

### Statistical analysis

Data obtained from the three replicates of the experiment are given as mean ± standard error mean (S.E.M.). One-way ANOVA (analysis of variance) with Tukey’s post hoc test was applied to examine the significance (*p* < 0.05) of each result. The complete data analytics was done with the help of SPSS software (version 20.0, SPSS Company, Chicago, USA). Pearson correlation analysis through PAST software (version 4.03) was conducted to identify significant relationships among various endpoint biomarkers associated with TCS-induced toxicity. Principal component analysis (PCA) was performed using Origin Pro (version 2024b).

## Results and discussion

The implications of sub-lethal TCS exposure on edible fish highlight the intricate dynamics between human actions and aquatic ecosystems. This study delves into the enduring effects of TCS on fish physiology, revealing the pervasive infiltration of this antimicrobial compound into aquatic environments. The 96-h LC_50_ of TCS was calculated as 0.742 mg/L using the Probit Analysis at 95% confidence limits. Almost similar findings were obtained in TCS-exposed *Oreochromis mossambicus*^22^.

The physicochemical parameters of the water, including pH, dissolved oxygen, hardness, temperature, and alkalinity, were within the recommended limits for fish survival^40^. Moreover, no significant fluctuations were observed in these parameters (Supplementary Table 2).

To assess the long-term effects of TCS, chronic toxicity experiments were performed using two concentrations below the LC_50_ value. During the chronic exposure, the fish displayed lesser activity, remaining stationary and showing movement only in response to disturbance. There were no instances of mortality throughout the chronic toxicity tests. Post-completion of stipulated experimental durations, liver was assessed for examining the TCS-induced toxicity as it offers a more instant evaluation of the xenobiotics contamination.

**Fig. 1:**
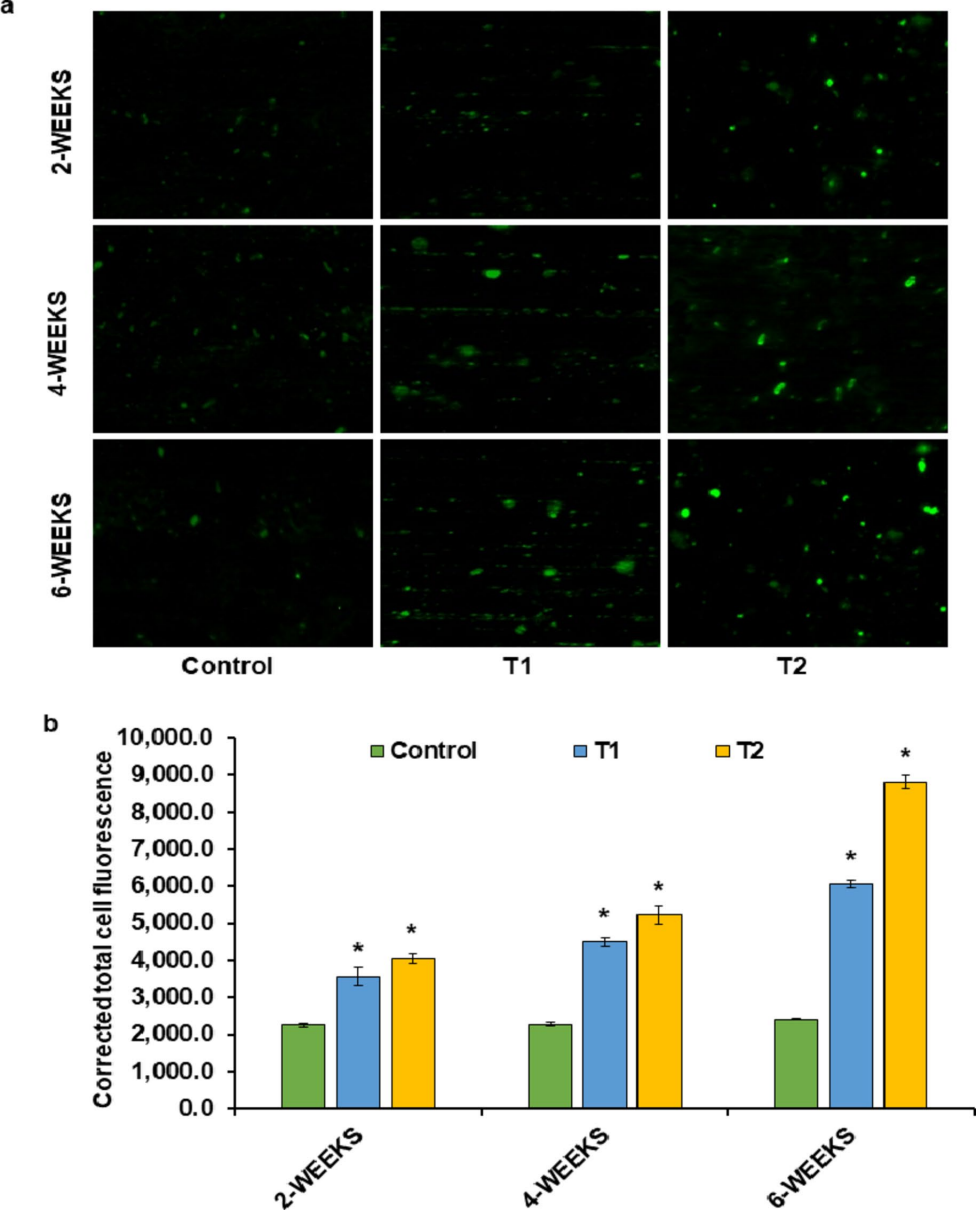
**a** Microphotographs showing fluorescent intensity of ROS level measured with DCFH-DA dye; **b** CTCF induced by TCS-exposure as compared to control at 2-, 4- and 6-weeks exposure. (mean ± S.E.M., *n* = 3 fish which were taken in triplicates); [*symbol represents the significant (*p* < 0.05) difference from the control, analyzed using one-way ANOVA with Tukey’s post hoc test].

ROS production is one of the most acceptable mechanisms to explicate toxicity caused by xenobiotics^54–57^. In this study, exposure to 0.0742 mg/L (T1) and 0.148 mg/L (T2) of TCS significantly (*p* < 0.05) elevated ROS levels in fish liver compared to control. The differences in fluorescent intensity of ROS are represented as microphotographs in Fig. 1a and the quantitated values are plotted in Fig. 1b.

After 2, 4, and 6 weeks of TCS exposure, we observed an alluring surge in the fold change of ROS production for T1 and T2, surpassing the control. Beginning with a modest fold change of 1.57 and 1.79 after 2 weeks, ROS levels increased to 1.97 and 2.29 at 4 weeks, culminating in a notable fold change of 2.51 for T1 and an impressive 3.65 for T2 after 6 weeks. The increase in ROS levels was dose-dependent over time, with T2 exhibiting the highest ROS production after 6 weeks. Our findings align with a previous study that reported a significant rise in ROS generation in *Danio rerio*embryos exposed to TCS, further supporting the oxidative stress-inducing effects of TCS^58^. Likewise, another research witnessed escalation in ROS production in the liver of *Larus michahellis*embryo after the exposure of TCS^59^. The TCS-induced ROS overproduction was also reported in the primary neurons of mice^60^. In the same way, several studies have recorded the heightened ROS generation in various human cell-lines following TCS treatment^61–64^. ROS encompasses a range of O_2_-derived entities such as: O_2_^•−^, H_2_O_2,_^•^OH; manifesting more reactivity than molecular oxygen^65^. ROS is one of the key-players of cellular physiology and biology^66^. These ‘double-sword’ entities, ROS, can disturb the redox balance of cells and lead to oxidative damage, if not effectively neutralized^67^.

**Fig. 2:**
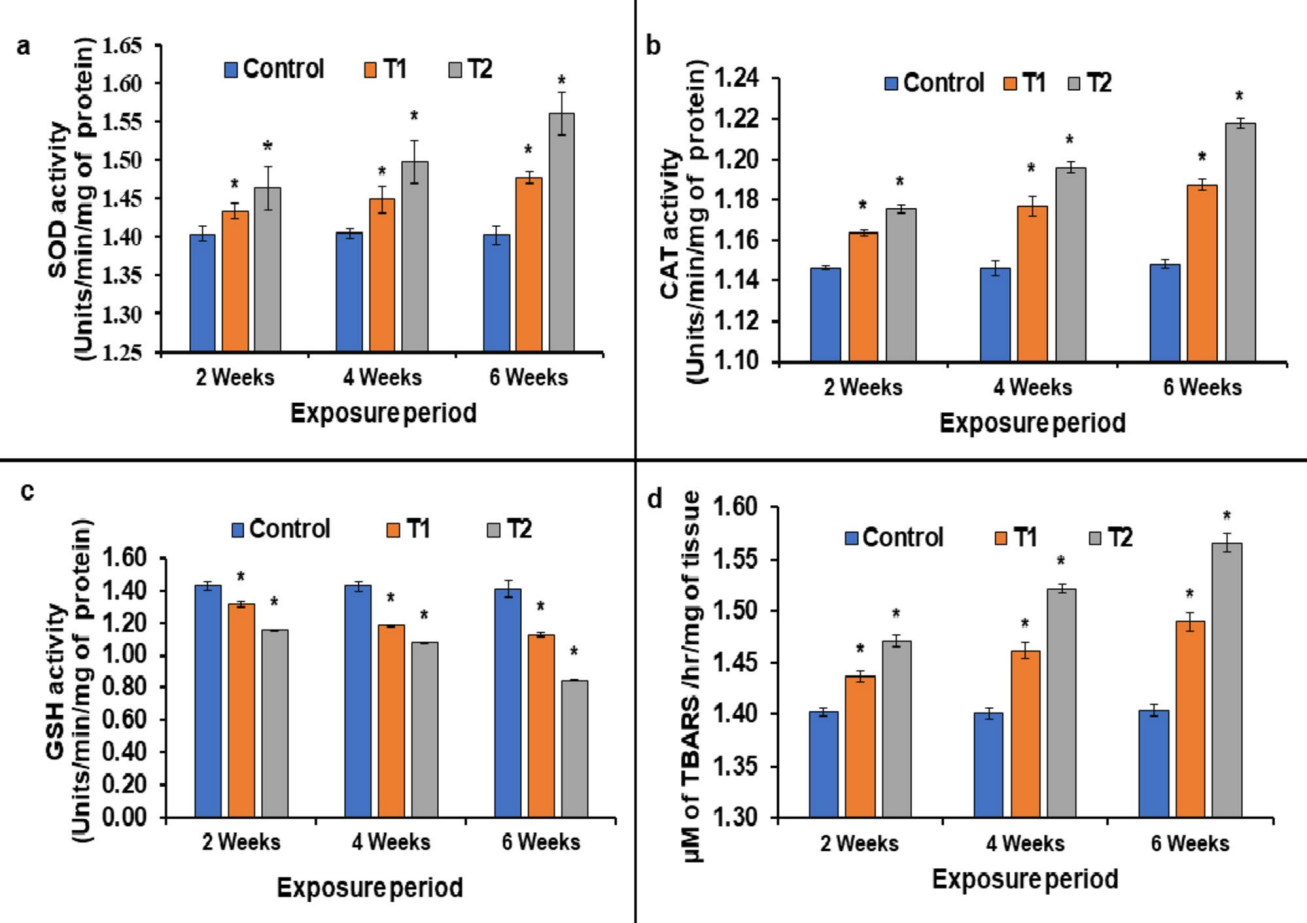
Activities of SOD (**a**), CAT (**b**), GSH (**c**) and LPO (**d**) in the liver of test fish *L. rohita* after 2, 4 and 6 weeks of exposure(mean ± S.E.M., *n* = 3 fish which were taken in triplicates); [*symbol represents the significant (*p* < 0.05) difference from the control, analyzed using one-way ANOVA with Tukey’s post hoc test].

To cope-up with the possible destructive impacts of ROS, the anti-oxidants (both enzymatic and non-enzymatic) defence machinery provides an adaptable mechanism to eliminate ROS, failure of which culminates into oxidative stress^68–71^. The state of ‘oxidative stress’ is characterized by the disruption of the dynamic balance between the generation of ROS and its removal by anti-oxidants^72,73^. Thus, assessing the extents of anti-oxidants is requisite to evaluate the degree of oxidative stress. The enzymatic antioxidant SOD initiates the removal of ROS by converting the superoxide radicals into H_2_O_2_ which is consecutively degraded into H_2_O and O_2_by CAT, which is another enzymatic anti-oxidant^74^. SOD and CAT are pivotal for stress resilience^75^, and an increment in their activities depicts the elevated oxidative stress levels. The present findings illustrated the significant (*p* < 0.05) rise in the activities of SOD and CAT in fish liver as represented in Fig. 2a and b, respectively.

The comparison of SOD levels in T1 and T2 with the control revealed fascinating dynamics over time. After 2 weeks, T1 exhibited a fold-change of 1.02, while T2 showed a fold-change of 1.04. Progressing to 4 weeks, both T1 and T2 displayed incremental rises, reaching 1.03 and 1.06 fold, respectively. The most notable increase occurred at the 6-week mark, with T1 and T2 elevating to 1.05 and 1.10 fold, respectively. Likewise, CAT activities in T1 and T2, contrasted with the control, demonstrated consistent patterns. At 2 weeks, T1 and T2 showcased marginal fold-change to 1.01 and 1.02, respectively. By 4 weeks, these increments in fold-change continued, reaching 1.02 and 1.04, respectively. This trend reached its peak at 6 weeks, when T1 and T2 increased to 1.03 and 1.06, respectively. The increase in both SOD and CAT was recorded to be duration- and dose-dependent with the highest upsurge observed in T2 after 6 weeks. Similar trends of augmentation in the SOD and CAT activities were reported in the liver of *Gambusia affinis*^76^; *L. rohita*^77^; *Pangasianodon hypophthalmus*^29^ and gills of *Ruditapes philipinnarum*^78^upon TCS-exposure. Elevated activities of SOD were also noted in the TCS-treated mice liver^79^. The outcomes of another research also validated that TCS-intoxication to adult Zebrafish led to the malfunctioning of the crucial first-line anti-oxidant defense system (i.e., SOD-CAT) in the liver^31^.

The second-line defense against oxidative stress is the non-enzymatic anti-oxidant system, primarily the glutathione system, of which GSH is the major component^80–82^. GSH performs a vital function in the removal of ROS but gets depleted during the execution of its role. Reduced GSH levels could enhance the cellular susceptibility to noxious agents, intensify the ROS-induced cytotoxicity and amplify the toxic potential of xenobiotics, eventually resulting in cell demise^83^. In this study, results displayed in Fig. 2c, depicts the significant (*p* < 0.05) decline in the levels of GSH in TCS-intoxicated fish liver. The evaluation of GSH levels, expressed as fold changes compared with control, revealed notable trends. At the 2-week mark, T1 and T2 showcased reductions to 0.92 and 0.81 fold, respectively. By the 4-week milestone, these reductions continued, yielding values of 0.83 and 0.75 fold. The most intriguing revelation emerged at 6 weeks, as T1 and T2 displayed remarkable reductions to 0.79 and 0.59 fold, respectively. With elevating dosage, the fall in GSH quantities followed a duration-dependent manner and T2 exhibited the most substantial decline after 6 weeks. Present outcomes were in tune with the results obtained after treating *L. rohita*, *Cyprinus carpio*,* Cirrhinus mrigal*,* Stenopharngodon idella*with TCS-quantities^84^. Similarly, TCS has also been reported to increase oxidative damage by reducing the GSH contents in *Anabas testudineus*^21^. In another study, the inhibition of GSH activity was reported in the lung tissue of TCS-treated rat^85^. Different human cell-types exposed to various TCS-concentrations displayed notable fluctuations in GSH extents^33,61,86^. In light of above-discussed findings, it was concluded that disrupted enzymatic and non-enzymatic anti-oxidants indicate a heightened state of oxidative burden.

Upon surpassing the cellular tolerance limit, ROS can destruct the membrane architecture, subsequently leading to lipid peroxidation^87^. The cataclysmic process of LPO can be initiated by ROS which consequently leads to the disruption of cellular and sub-cellular membranes by altering their integrity, fluidity and permeability; as well as impacting the activity of lipid-anchored proteins^88^. Since, polyunsaturated fatty acids (PUFAs) are easily degraded upon oxidation, the products of LPO are a valuable indicator to delineate oxidative damage^65^. LPO is often quantified by measuring end-products such as 4-hydroxynonenal (HNE) and malonyldialdehyde (MDA)^89^. In present study, the reaction of MDA with thiobarbituric acid reactive substance (TBARS) was used to evaluate LPO. The outcomes of our experimentation clearly evinced that TCS-exposure led to significant (*p*< 0.05) elevations in the LPO levels. Throughout 2, 4, and 6 weeks, the fold-changes in LPO quantities for T1 and T2, juxtaposed against the control, unveiled a progressive trend. There were modest fold-change of 1.02 and 1.05, respectively, after 2 weeks. This subtle upsurge continued its ascent at 4 weeks, marking fold-change of 1.04 for T1 and 1.08 for T2. After 6 weeks the fold-changes reached their peak with pronounced escalations to 1.06 for T1 and a notable 1.11 for T2, echoing the profound dynamics of cellular adaptation. The increment in LPO levels was influenced by both exposure-duration and administered-dosage; with the most remarkable rise noted in T2 after 6 weeks. The alterations in LPO activity were displayed in the form of TBARS levels as illustrated in Fig. 2d. Here-acquired findings were in accordance with the outcomes of Hemalatha (2019)^90^ who observed elevations in LPO contents in liver, gill and kidney of TCS-treated *L. rohita.* Similar increasing patterns of LPO were noticed in the various organs of TCS-exposed *Anabas testudineus*^21^; *Carassius auratus*^91^; *Danio rerio*^31^; *Larus michahellis*^59^; *Ruditapes decussatus*^92^; and *Solea senegalensis*^93^. Likewise, other studies also pointed out the increased levels of LPO in Sprague-Dawley rats^94,95^; domestic goat^96^and indifferent human cell lines^61,86,97^, after being intoxicated with varied doses of TCS.

**Fig. 3:**
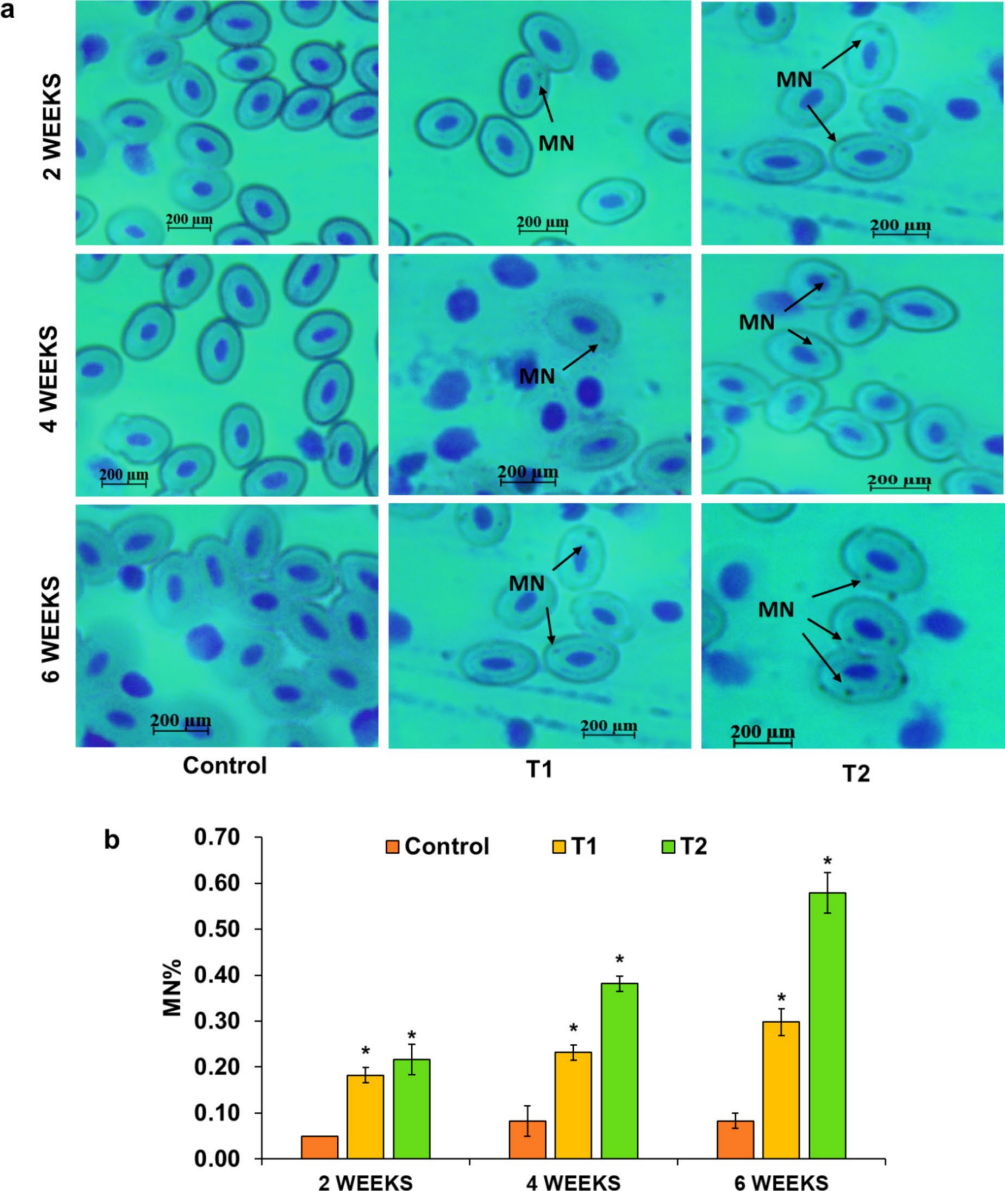
**a** Microphotographs showing MN and (**b**) MN frequency induced by TCS in T1 and T2 compared to control for 2, 4, and 6 weeks. [*symbol represents the significant (*p* < 0.05) difference from the control, analyzed using one-way ANOVA with Tukey’s post hoc test].

ROS overproduction relentlessly targets DNA, causing extensive damage that shatters the integrity of genetic material. As oxidative stress escalates, ROS-driven LPO produces DNA-reactive lesions, amplifying the destruction and pushing cells toward genomic instability^98^. The MN assay is a prominent technique for identifying DNA damage. It has been extensively utilized in research to examine chromosomal alterations in vivo, serving as a vital approach for evaluating DNA stability after exposure to radiation, hazardous chemicals, and other environmental stressors^99–101^. A rise in MN frequency serves as a clear marker of chromosomal damage. The present study demonstrated that TCS exposure led to a dose- and time-dependent significant (*p* < 0.05) elevation in micronucleus frequency, as illustrated in Fig. 3. Comparison of MN frequency in T1 and T2 with the control highlighted distinct patterns over time. After 2 weeks, T1 showed a 3.71-fold change, while T2 showed a 4.38-fold change. At 4 weeks, T1 and T2 had fold changes of 2.81 and 4.64, respectively. By 6 weeks, T1 reached a 3.62-fold change and T2 reached a 7.06-fold change, with the maximum increment observed in T2. Aligning with our results, TCS intoxication markedly increased DNA damage and micronucleus frequency in the liver and blood cells of *Pangasianodon hypophthalmus*^102^. Similar findings were reported in *Clarias gariepinus*, where MN frequency rose with TCS exposure^103^. Similarly, *L. rohita*exhibited heightened MN levels after TCS treatment^104^, reinforcing the conclusion that TCS induces DNA damage.

It was well-confirmed with the above outlined observations that exposure to TCS instigated the overproduction of ROS, resulting in disrupted antioxidant activity and subsequent degradation of cellular membranes via lipid peroxidation. These combined outcomes underscore the troubling existence of oxidative trauma within the livers of the treated fish. Enhanced oxidative stress elicits a plethora of adverse effects, from harming cellular components to inducing cell death. Moreover, it can also interfere with the functionality of redox-sensitive cellular organelles, notably the mitochondria and ER^105^.

ER, being an indispensable organelle, is responsible for crucial functions such as detoxification of toxic substances; calcium storage; lipid production; and maintaining proteostasis i.e., the synthesis, appropriate folding, translocation and post-translational modifications of protein^106^. For effective protein folding within the ER, high contents of calcium are necessary to aid the activity of ER chaperones^107^. TCS has been well-documented to impair calcium equilibrium^108^. It triggers the release of Ca^2+^from intracellular stores by raising the membrane permeability, depleting calcium within the ER and consequently elevating intracellular calcium levels^109^. The dysregulation of calcium homeostasis subsequently causes misfolded/unfolded proteins to accumulate inside the ER^110^. In our study, we observed a time- and dose-dependent increase in intracellular calcium content in the liver (given in Supplementary Fig. 1). The fold change in calcium concentrations was assessed in T1 and T2 relative to the control. At 2 weeks, the fold change was 1.23 in T1 and 1.37 in T2. By 4 weeks, the fold change increased to 1.37 in T1 and 1.50 in T2. After 6 weeks, the fold change further rose to 1.49 in T1 and 1.69 in T2. The findings clearly demonstrated that TCS increased intracellular calcium, which drained calcium from the ER, led to protein misfolding and the onset of ER-stress.

Furthermore, the elevated oxidative stress can exacerbate the build-up of mal-folded/ unfolded polypeptides in the lumen of ER which eventually creates the environment of distress that is termed as ER-stress^105^.

To sustain the ER-proteostasis and counteract the enhanced ER-stress, the adaptive mechanisms are activated which includes ER-associated degradation (ERAD), reticulophagy and unfolded protein response (UPR)^13^. ERAD regulates the homeostatic abolition of misfolded/non-folded proteins from the ER-lumen to overcome ER-stress^110^. Reticulophagy, a process that involves the remodelling of ER and its selective recycling via autophagy, mitigates the ER-stress by eradicating surplus membranes and proteins^111^. Further, the evolutionarily conserved ‘UPR’ serves as a precisely orchestrated network of signal pathways being exec uted intracellularly to regain the ER-proteostasis^112^. Among the afore-mentioned adaptive responses, we particularly targeted the UPR to investigate the processes involved in combating ER-stress. The major pathways of UPR are controlled by three key trans-membrane proteins present in ER viz. IRE1 (inositol-requiring enzyme 1), ATF6 (activating transcription factor 6) and PERK (protein kinase R-like endoplasmic reticulum kinase)^19^. In normal physiological conditions, the master regulator ER-chaperone GRP78 (glucose-regulated protein 78) binds to the luminal ends of IRE1, ATF and PERK to keep them in “off” state^106^. Since GRP78 (also known as BiP) favourably binds to unfolded proteins, the accumulation of unfolded polypeptides in the ER lumen disrupts the association of GRP78 with IRE1, ATF6 and PERK; allowing conformational changes in these trans-membrane proteins to switch them “on”^105^. As UPR involves three trans-membrane proteins that trigger distinct signaling cascades, our study specifically focuses on the PERK pathway to explore the underlying mechanisms of the UPR.

PERK is an essential type-1 trans-membrane protein, belonging to the distinguished family of eukaryotic translation initiation factor 2α (eIF2α) kinases. Under ER-stress, PERK dimerizes after GRP78 dissociation, and undergoes trans-autophosphorylation, leading to the activation of its intracellular kinase domain. Activated PERK phosphorylates Ser-51 on eIF2α, blocking its recycling into the active GTP-bound form required for nascent protein synthesis. This disruption prevents the transfer of initiator methionyl-tRNA to the translation initiation complex, effectively attenuating the cap-dependent translation^113^. Although the translational block is widespread, it’s not entirely prohibitive. Certain genes, like activating transcription factor 4 (ATF4), can still be translated selectively in these circumstances, facilitated by the presence of upstream open reading frames and internal ribosomal entry sites^114^. ATF4, the transcription factor, migrates to the nucleus, initiating the transcription of genes essential for alleviating ER stress^115^. The ATF4-driven expression of CCAAT/enhancer binding-homologous protein (CHOP) is heightened, representing a crucial pro-apoptotic transcription factor. Studies indicate that CHOP facilitates apoptosis via various pathways, including the mitochondrial-dependent pathway, death receptor pathway, and additional routes^116^. In our study, we meticulously scrutinized the relative expression of UPR-related genes. Comparing them to the control group, we evaluated mRNA expression at both T1 and T2, six weeks apart, presenting our findings in fold change: *grp78* exhibited a fold change of 4.31 ± 0.52 at T1 and 12.90 ± 1.16 at T2, *perk* showed 3.59 ± 0.22 at T1 and 10.62 ± 0.71 at T2, *eIF2α* displayed 12.12 ± 1.97 at T1 and 22.10 ± 0.93 at T2, *atf4* demonstrated 8.53 ± 0.91 at T1 and 12.74 ± 1.96 at T2, while *chop* presented 5.36 ± 0.48 at T1 and 8.5 ± 1.01 at T2. These findings offer compelling insights into the dynamic responses of these genes over time. The heightened expression of ER-stress related genes obtained here confirmed the activation of the PERK/eIF2α/ATF4/CHOP pathway, validating the induction of UPR in the liver of TCS-exposed fish. The PERK pathway plays a crucial role in reducing the load of newly synthesized proteins entering the ER, thereby allowing the cell to manage and resolve the accumulation of misfolded proteins more effectively. By examining the PERK signaling cascade, we aimed to elucidate how cells modulate protein synthesis, enhance the expression of stress-related genes, and ultimately achieve ER-homeostasis under conditions of stress via activation of UPR.

**Fig. 4:**
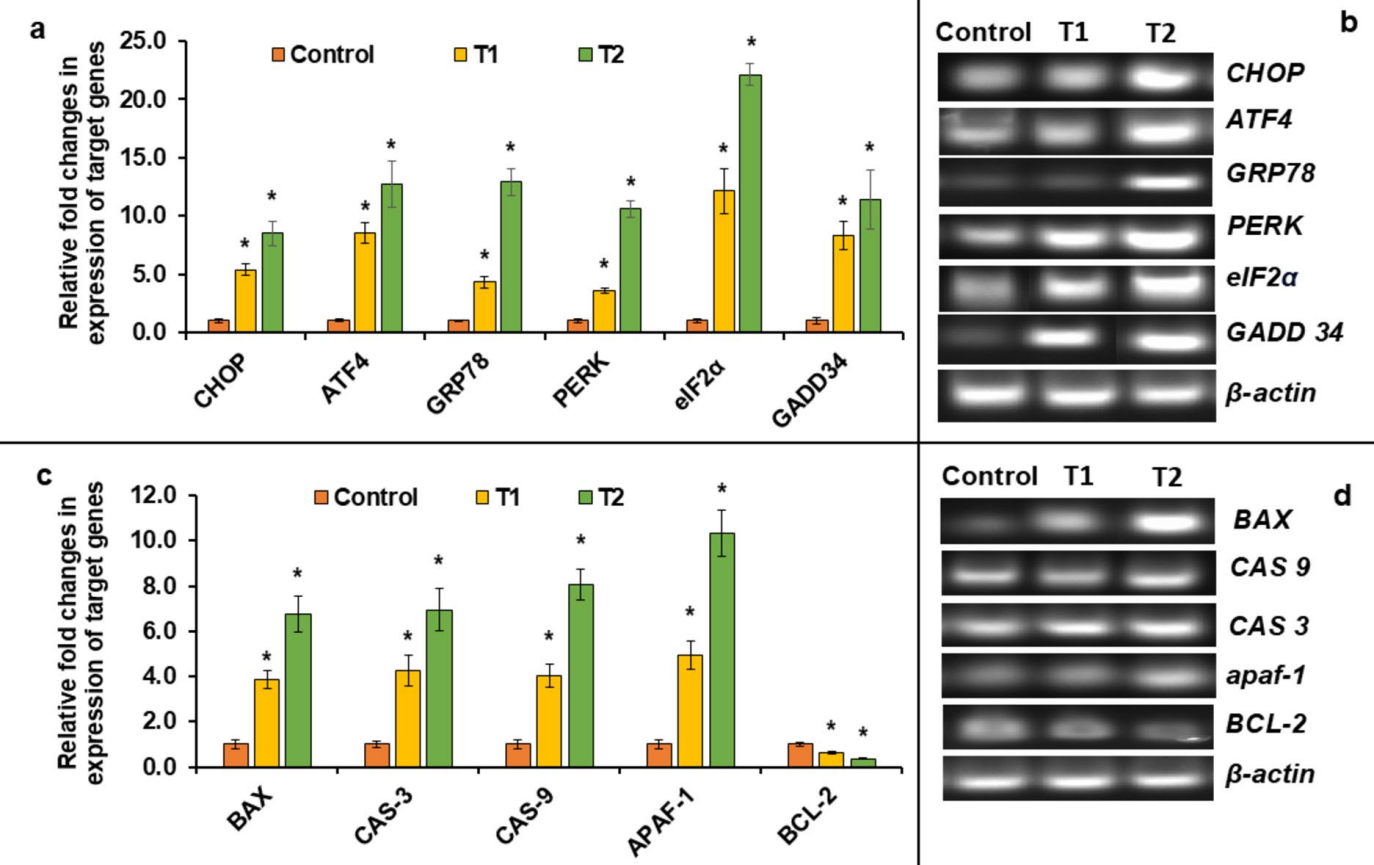
**a**, **c** Represent qRT-PCR analysis of the expression of ER-stress and apoptosis-related genes, respectively; while (**b**) and (**d**) show the band intensity of the ER-stress and apoptotic genes, respectively, normalized with ß-actin. [mean ± S.E.M, *n* = 3 fishes (triplicates)]. [*symbol represents the significant (*p* < 0.05) difference from the control, analyzed using one-way ANOVA with Tukey’s post hoc test].

The adaptive UPR effectively diminishes the unfolded protein burden within the ER, preserving cell viability and function. Yet, prolonged activation of the UPR can ultimately lead to cell death^19^. Elevated CHOP expression has been associated with a transition towards pro-apoptotic outcomes, achieved through direct regulation of downstream pro-apoptotic target genes and indirectly by reinitiating cap-dependent translation via the induction of growth arrest and DNA damage-inducible protein 34 (GADD34), which in turn de-phosphorylates eIF2α^117^. The subsiding of translational blockage adds to the build-up of unfolded proteins in the ER-compartment while also allowing for the translation of mRNAs encoding pro-apoptotic proteins^112^. Among its various functions, CHOP specifically triggers cell apoptosis by directly modulating the expression of B-cell lymphoma protein 2 (BCL-2) and enhancing the BCL-2-associated X protein (BAX) expression^106^. A counterpart of BCL-2 collaborates with Apoptotic protease activating factor-1 (APAF-1) to prevent the activation of allied caspases. Similarly, BCL-2 functions to inhibit apoptosis by partially obstructing the mitochondria from releasing cytochrome-c^118^. However, when BCL-2 is down-regulated and BAX is up-regulated, it leads to the release of cytochrome-c from the mitochondria, followed by the activation of APAF-1. Cytochrome-c teams up with PROCASPASE-9 and APAF-1 to create the apoptosome, a crucial complex in the cell. This powerful formation triggers the auto-activation of CASPASE-9, which then turns-on the effector CASPASE-3, driving the cell towards apoptosis^119,120^. The mRNA expression of *gadd34* surged by an impressive 45.8 ± 1.20 and 11.40 ± 2.51 fold, indicating a substantial upregulation in T1 and T2 compared to control group after 6 weeks. Similarly, *bax* exhibited a profound increase, with fold-changes of 3.87 ± 0.41 and 6.75 ± 0.79 in T1 and T2, respectively. The expression of *apaf-1* also showed a noteworthy rise, reaching 14.93 ± 0.62 and 10.34 ± 1.01 fold in T1 and T2. Notably, *caspase-9* displayed a marked elevation of 4.03 ± 0.50 and 8.05 ± 0.68 fold, while *caspase-3* exhibited increases of 4.24 ± 0.67 and 6.95 ± 0.95 fold in T1 and T2 against the control. Conversely, the expression of anti-apoptotic *bcl-2* showed a remarkable decrease, with fold-changes of 0.63 ± 0.03 and 0.35 ± 0.02 in T1 and T2, respectively, juxtaposed to control after 6 weeks. The shifts in the expression levels of ER-stress and apoptosis-related genes were statistically significant (*p* < 0.05), as depicted in Fig. 4a and c, respectively. Quantitative analysis of DNA band densitometry was conducted for ER stress-related genes including *CHOP*,* ATF4*,* GRP78*,* PERK*,* eIF2α*, and *GADD34* (visualized in Fig. 4b), as well as apoptosis-related genes like *BAX*,* CASPASE-3*,* CASPASE-9*,* APAF-1*, and *BCL-2* (depicted in Fig. 4d) in liver tissue sample of all experimental groups.*β-actin*, serving as a housekeeping gene, was utilized as a reference for data normalization. The full-length images of gel bands are given in supplementary information file [Supplementary Fig. 2(a-m)].

Our findings clearly demonstrated that TCS-induced ER-stress eventually resulted in the death of fish hepatocytes. A similar research on triclocarbon-exposed zebrafish reported ER-stress induction and subsequent activation of UPR^121^. Further, another research stated that ER stress-mediated apoptotic key markers such as caspase-3, GRP78 and CHOP were highly expressed in the liver of copper-treated mice^106^. In the same way, the altered expression levels of apoptosis-related genes was reported in the gill and ovary of TCS-intoxicated *Danio rerio*^122^. Likewise, arsenic-stressed environment, eventually evoked PERK/ IRE-1 stimulated apoptosis in the spleen of *Cyprinus carpio*^52^.

**Fig. 5:**
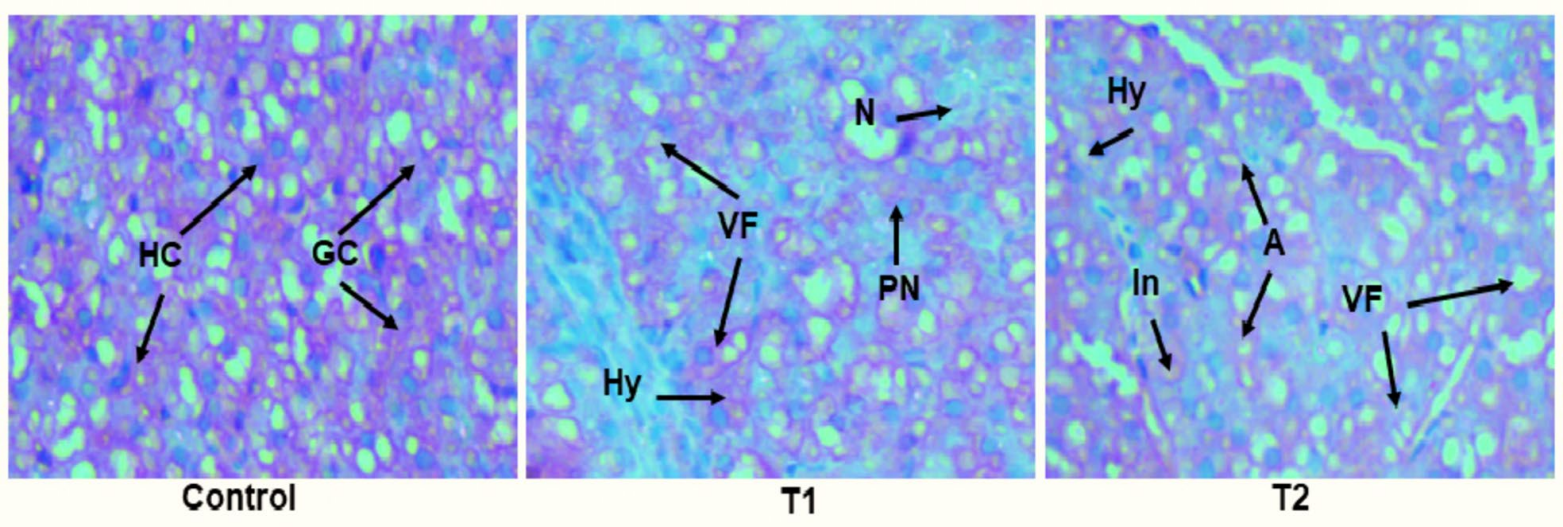
Microphotographs showing histological alterations in the liver of test fish L. rohita after 6 weeks. (HC-Hepatic cells; GC-Granular cytoplasm; N-necrosis; Hy-Hypertrophy; PN-Pyknosis; VF-Vacuole formation; In-Inflammation; A-Apoptosis).

The exposure to TCS and its consequent toxicity, characterized by heightened oxidative stress and ER stress-induced programmed cell death, resulted in severe histological damage in the liver of experimental fish. The effect of prolonged TCS intoxication on the hepatic structure of test fish after 6 weeks is portrayed in Fig. 5. The histological structure of the unexposed liver displayed healthy hepatic cells (HC) and granular cytoplasm (GC). In T1, substantial alterations were observed, including necrosis (N), hypertrophy (Hy), pyknosis (PN), and vacuole formation (VF). Meanwhile, T2 exhibited notable deformities such as vacuole formation, hypertrophy, inflammation (In), and apoptosis (A).

**Fig. 6:**
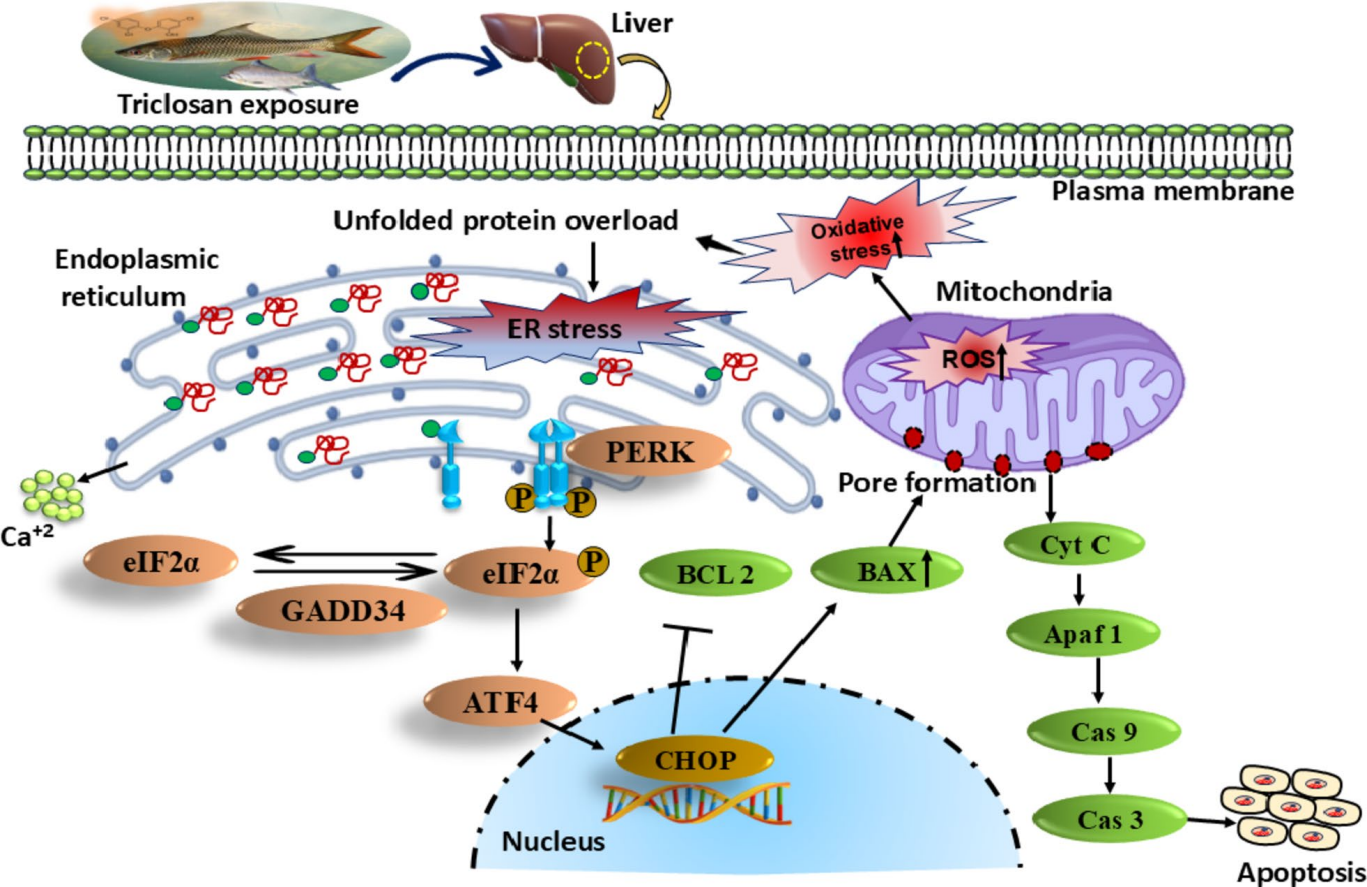
Schematic representation of the PERK/eIF2α/ATF4/CHOP signaling cascade triggered by oxidative stress and ROS-induced ER-stress in the liver of food fish *Labeo rohita*. The diagram highlights the interplay between UPR and apoptotic pathways, leading to hepatocyte apoptosis after exposure to sub-lethal concentrations of Triclosan.

Our findings were in tune with a previous finding which reported necrosis and atrophy in the liver of TCS-intoxicated zebrafish^31^. Likewise, in another similar research conducted on *Anabas testudineus*, several histological perturbations like vacuolization, elongated nucleus and degenerated cytoplasm were documented in the liver after chronic exposure to TCS^21^. Herein, we explored the damaging potential of TCS at various levels and, most notably, unveiled a novel mechanism that links the UPR and apoptosis signaling pathways in the liver of *L. rohita* exposed to sub-lethal concentrations of TCS (Fig. 6).

**Fig. 7:**
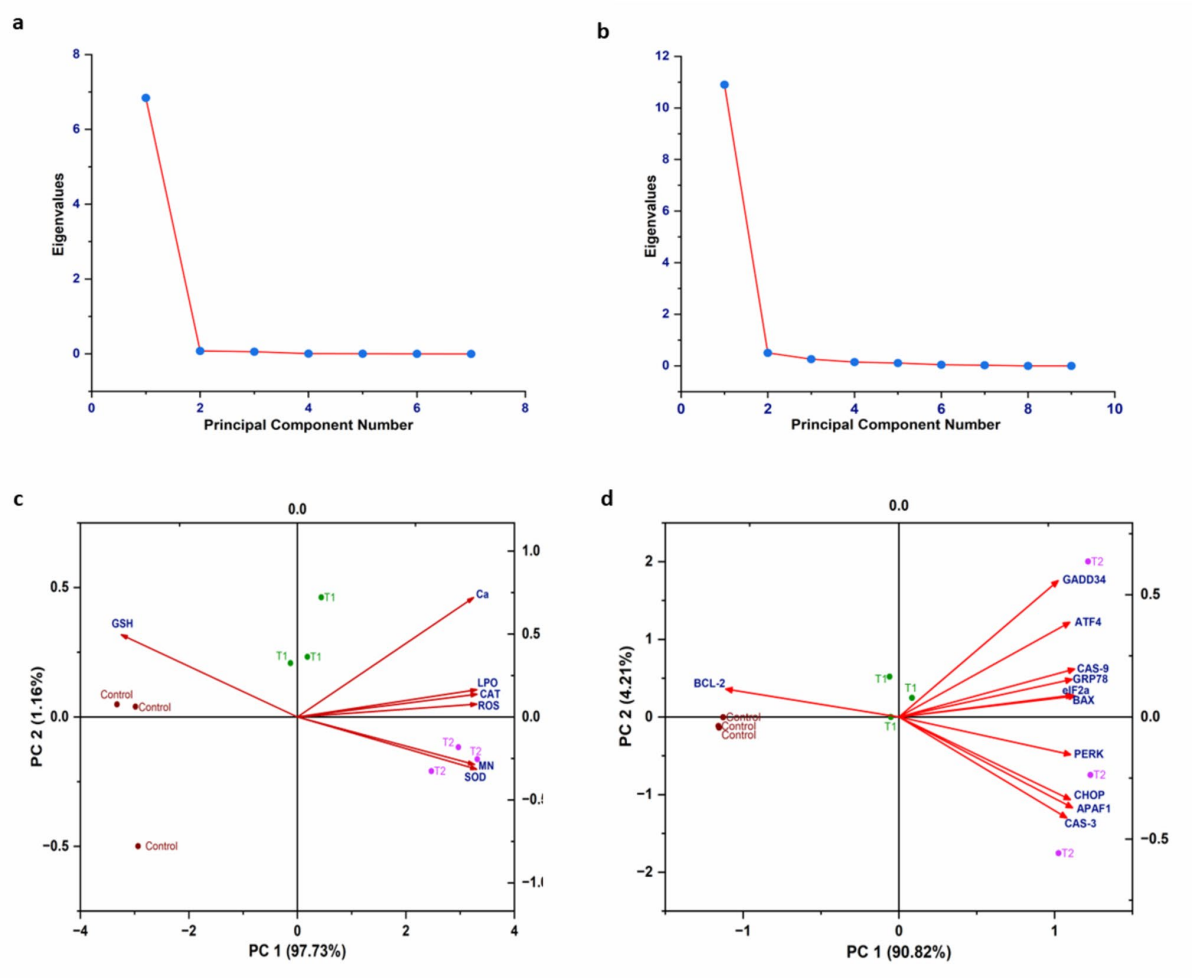
**a** Scree plot and (**c**) Bi-plot of PCA for oxidative stress-related parameters (ROS, SOD, CAT, GSH, and LPO) and physiological indicators (Ca levels and MN frequency); while (**b**) Scree plot and (**d**) Bi-plot of PCA for genes associated with ER-stress and apoptosis.

PCA was performed to transform the original variables into a smaller set of derived variables, simplifying interpretation by capturing the maximum variability in the data^123^. The variance of oxidative stress parameters, MN%, Ca, and genes associated with ER-stress and apoptosis on PCA is delineated in Table 1. Figure 7a presents a scree plot illustrating the eigenvalues of factors related to oxidative stress parameters (ROS, SOD, CAT, GSH, and LPO) alongside physiological indicators (Ca levels and MN frequency), while Fig. 7b displays a scree plot for genes associated with ER stress and apoptosis. In Fig. 7c, PCA showed that 98.89% of the total variance was explained by the first two principal components (PCs). PC1 accounted for 97.73% of the variance, with GSH being the only variable making a negative contribution. PC2 explained 1.16% of the total variance and had a positive correlation with variables such as SOD, CAT, LPO, MN%, and Ca. In Fig. 7d, PCA accounted for 95.03% of the total variance including PC1 and PC2. PC1 explained 90.82% of the variance, with BCL-2 as the only negatively contributing variable. PC2 accounted for 4.21% of the variance, showing a positive correlation with variables such as GADD34, ATF4, CAS-9, GRP78, eIF2α, BAX, PERK, CHOP, Apaf1, and CAS-3.

**Table 1:** The interpretation of variance of oxidative stress parameters, MN%, Ca, ER stress and apoptotic genes on principal component analysis.

Parameters	PC1	PC2
ROS	0.38046	0.07683
SOD	0.38041	− 0.31574
CAT	0.3813	0.137
GSH	− 0.3738	0.49633
LPO	0.38048	0.16506
MN%	0.3756	− 0.2873
Ca	0.3736	0.72077
CHOP	0.30125	− 0.34389
ATF4	0.30081	0.38187
GRP78	0.30439	0.14924
PERK	0.30148	− 0.15797
eIF2α	0.30984	0.08229
GADD34	0.28028	0.55309
BAX	0.30514	0.08419
CAS-3	0.29462	− 0.41952
CAS-9	0.30883	0.19184
APAF-1	0.30474	− 0.37691
BCL-2	− 0.30416	0.11859

**Fig. 8:**
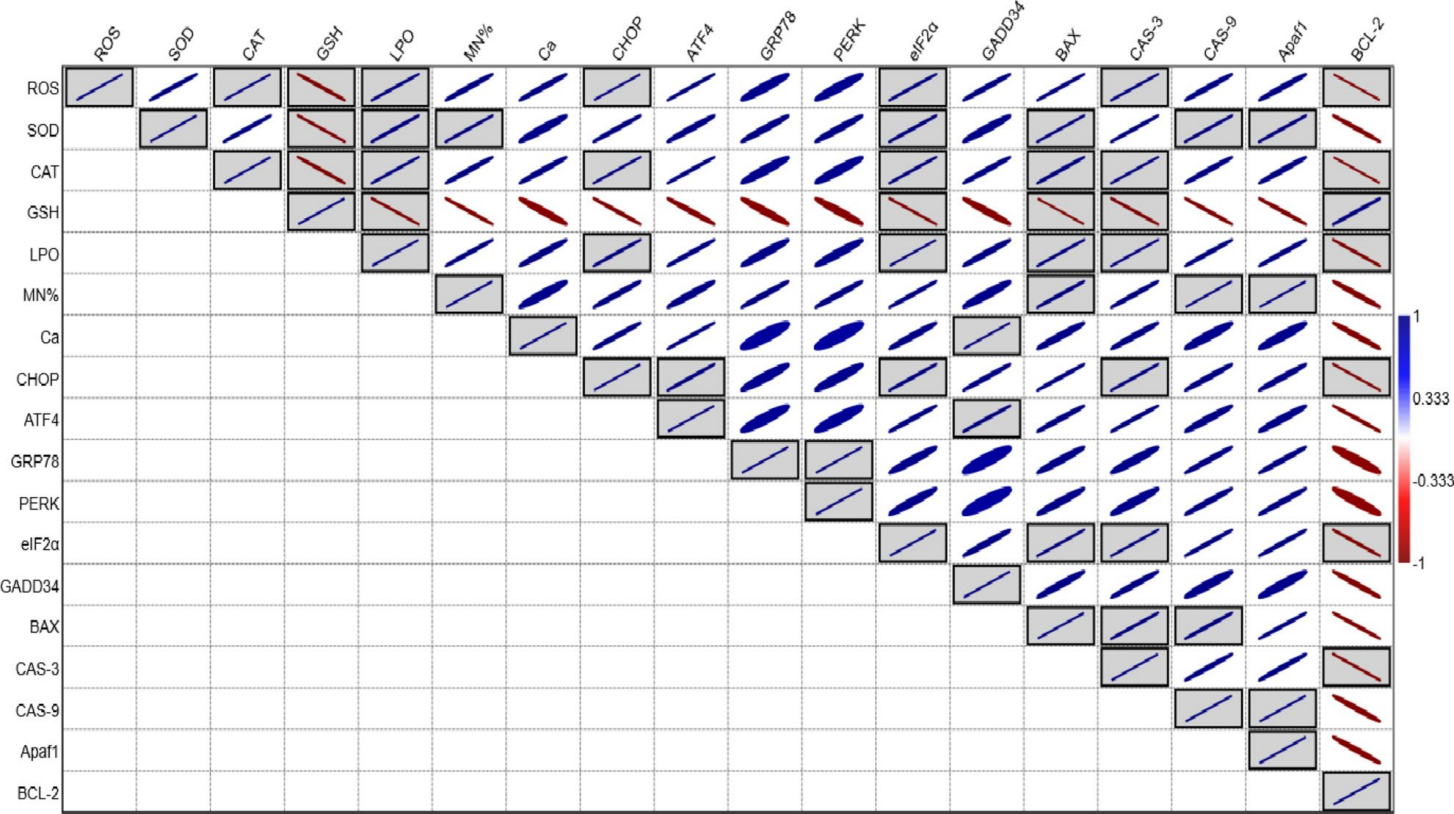
Pearson correlation matrix depicting the significant relationships between various parameters associated with TCS-induced toxicity in *Labeo rohita*.

The Pearson correlation analysis uncovered several significant associations among the studied parameters, reflecting intricate interrelationships (as represented in Fig. 8). ROS was found to be significantly (*p* < 0.05) positively correlated with CAT, LPO, CHOP, eIF2α, and CAS-3. Likewise, SOD exhibited significant (*p* < 0.05) positive correlations with LPO, MN%, eIF2α, BAX, CAS-9, and APAF-1. CAT also showed significant (*p* < 0.05) positive correlations with ROS, LPO, CHOP, eIF2α, BAX, and CAS-3. In contrast, GSH had a significant (*p* < 0.05) negative correlation with ROS, SOD, CAT, LPO, eIF2α, BAX, and CAS-3, while showing a positive correlation with Bcl-2. LPO exhibited a significant (*p* < 0.05) positive correlation with ROS, SOD, CAT, CHOP, eIF2α, BAX, and CAS-3, whereas MN% was significantly (*p* < 0.05) positively correlated with SOD, BAX, Cas-9, and APAF-1. Calcium levels showed a significant (*p* < 0.05) positive correlation with GADD34. CHOP displayed significant (*p* < 0.05) positive correlations with ROS, CAT, LPO, ATF4, eIF2α, and CAS-3. ATF4 was significantly (*p* < 0.05) positively correlated with both CHOP and GADD34. The analysis also revealed a significant (*p* < 0.05) positive correlation between GRP78 and PERK, while eIF2α was significantly (*p* < 0.05) positively correlated with ROS, SOD, CAT, LPO, CHOP, BAX, and CAS-3. BAX significantly (*p* < 0.05) correlated positively with SOD, CAT, LPO, MN%, CAS-3, and CAS-9, while CAS-9 exhibited significant (*p* < 0.05) positive correlations with SOD, BAX, and APAF-1 Interestingly, Bcl-2 demonstrated a significant (*p* < 0.05) negative correlation with ROS, CAT, LPO, CHOP, eIF2α, and Cas-3, but a positive one with GSH. These findings underscore the complex and intertwined relationships between oxidative stress, ER-stress, apoptosis, and the cellular defense systems.

## Conclusion

Our findings offer compelling validation that exposure to TCS elicited ROS production, disrupted the antioxidant defence machinery, compromised DNA integrity and subsequently triggered the PERK pathway through the activation of ER-stress response mechanisms, as part of the UPR. This intricate cascade ultimately led to the demise of hepatocytes in *L. rohita*, a species extensively consumed by humans. Any toxic effects on this food fish can directly impact human health via food chain. Remarkably, the post-COVID-19 era has seen a surge in TCS pollution due to heightened use of disinfectants and antimicrobial products, amplifying environmental and health risks. Although present environmental TCS concentrations are below sub-lethal doses, the potential for contamination to rise is concerning. As TCS concentrations rising at an alarming rate due to extensive disinfectant use, it is inevitable that they will soon breach critical sub-lethal thresholds, unleashing devastating consequences for aquatic ecosystems and putting human health at grave risk. Our findings illuminate the molecular mechanisms behind TCS-induced hepatotoxicity and underscore the urgent need to understand ER-stress pathways in the context of rising TCS contamination in environmental toxicology.

## Electronic supplementary material

Below is the link to the electronic supplementary material.

Supplementary Material 1.

## Data Availability

All data supporting the findings of this study were generated during the research detailed in this manuscript (and its Supplementary Information files).

## Abbreviations

TCS: Triclosan
ER: Endoplasmic reticulum
cDNA: Complementary deoxyribonucleic acid
UPR: Unfolded protein response
IRE-1α: Inositol requiring enzyme-1α
ATF: Activating transcription factor
PERK: Protein kinase-like endoplasmic reticulum kinase
LC: Lethal concentration
DMSO: Di-methyl sulphoxide
KMnO_4_: Potassium permanganate
CaCO_3_: Calcium carbonate
DO: Dissolved oxygen
TDS: Total dissolved solids
APHA: American Public Health Association
OECD: Organisation for economic co-operation and development
ROS: Reactive oxygen species
DCFH-DA: Di-chlorodihydrofluorescein diacetate
MN: Micronucleus
PBS: Phosphate buffered saline
SOD: Superoxide dismutase
CAT: Catalase
GSH: Reduced glutathione
LPO: Lipid peroxidation
Ca: Calcium
ICP-MS: Inductively coupled plasma mass spectrometry
NBF: Neutral buffered formalin
DPX: Dibutylphthalate polystyrene xylene
qRT-PCR: Quantitative real-time polymerase chain reaction
CHOP: CCAAT/enhancer binding-homologous protein
GADD34: Growth arrest and DNA damage-inducible protein 34
eIF2α: Eukaryotic initiation factor 2α
GRP78: Glucose-regulated protein 78
BAX: Bcl-2 associated X protein
BCL-2: B-cell lymphoma-2
APAF1: Apoptotic protease activating factor-1
PUFA: Polyunsaturated fatty acid
MDA: Malonyldi-aldehyde
TBARS: Thiobarbituricacid reactive substance
HNE: 4-hydroxynonenal
mRNA: Messenger ribonucleic acid
CAS: Caspase
PCA: Principal component analysis
