# Supplementary material for: Unveiling the crosstalk between unfolded protein response and apoptosis in triclosan induced hepatotoxicity in Labeo rohita
Source: Sci Rep. 2025 May 16;15:17089. doi: 10.1038/s41598-025-93997-0 (PMC12084320; doi:10.1038/s41598-025-93997-0)
Supplement: Supplementary file 1 — Supplementary Material 1. [file 41598_2025_93997_MOESM1_ESM.pdf]

Unveiling the crosstalk between Unfolded Protein Response and Apoptosis in Triclosan induced hepatotoxicity in *Labeo rohita*

Name of Gene	Primer Sequence	Product length	Tm (°C)	Accession number
CHOP	(F) ACAGGAGGACACGTAGAG<br>(R) CACTAAACCTCCCTTTCTCC	129 bp	54.89<br>55.06	NM_001082825.1
PERK	(F) GGAACAGGGATAGAGAGAGT<br>(R) CAGCTTACGAGGTGTCAT	657 bp	55.05<br>54.00	BC122104.1
GADD34	(F) AACCTTTATTCGTCCAGTCC<br>(R) TTCCTCCCAACACAAATGAA	193 bp	55.07<br>55.05	NM_001082921.1
eIF2α	(F) TGTGAGTCTGTGTTCGATTT<br>(R) ATCCCAAACTTCCATCTGTC	120 bp	54.96<br>55.02	NM_001025170.2
GRP78	(F) TCTACAAGAATGGACGTGTT<br>(R) TTTTCAGGGTTGGATGTTAGA	139 bp	54.34<br>54.73	AW421612.1
ATF4	(F) CCAGAGCTAAACCATACTCC<br>(R) TACAAGCACTACAGCATCAG	490 bp	54.94<br>55.20	NM_213233.1
BAX	(F) GGCAATGACCAGATACTTGA<br>(R) TCGGCTGAAGATTAGAGTTG	232 bp	55.13<br>54.91	AF231015.1
BCL-2	(F) TTCAAAGCGAGGATATGTGT<br>(R)ATCAGGCATTCAGAGTTGTT	147 bp	54.77<br>54.93	AY695820.1
CASPASE-9	(F) GAAATACAGAGCAAGGCAAC<br>(R) CAGTGGTACTAATCGTGGAG	273 bp	54.97<br>55.02	MG958002.1
CASPASE-3	(F) GAACTTTGATCGCAGGACAG<br>(R) TAGAGGAAGTCTGCTTCAACCG	432 bp	56.83<br>59.77	AB047003.1
APAF1	(F) ATGAACTCATACCATGCGG<br>(R) ATCTTCTTCAGATTGCCTCC	155 bp	55.05<br>54.81	AF251502.1
β-actin	(F) GTATTGTCACCAACTGGGAT<br>(R) ATACCGCAAGATTCCATACC	601 bp	55.03<br>54.99	AF025305.1

Supplementary Table-1: Primer pair sequences of ER stress and Apoptotic genes for qRT-PCR.

Physicochemical Parameters	Groups	Exposure Period		
		2 Weeks	4 Weeks	6 Weeks
**Alkalinity** (as $CaCO_3$ mg l^{-1})	**Control**	74.64±0.01	73.75±0.01	77.16±0.01
	**T1**	74.13±0.01	75.13±0.01	77.67±0.01
	**T2**	75.50±0.05	76.79±0.01	74.66±0.08
**DO** (mg l^{-1})	**Control**	7.38±0.16	7.05±0.02	6.81±0.11
	**T1**	6.44±0.02	7.24±0.01	6.85±0.01
	**T2**	7.50±0.01	6.44±0.01	6.50±0.091
**Hardness** (as $CaCO_3$ mg l^{-1})	**Control**	73.31±0.01	74.07±0.01	74.49±0.01
	**T1**	73.77±0.01	75.13±0.01	74.96±0.68
	**T2**	73.46±0.01	74.78±0.01	74.23±0.01
**pH**	**Control**	7.19±0.01	7.17±0.01	7.21±0.01
	**T1**	7.27±0.01	7.10±0.11	7.18±0.02
	**T2**	7.24±0.01	7.13±0.17	7.04±0.12
**Temperature** (°C)	**Control**	24.60±0.15	23.44±0.02	24.48±0.01
	**T1**	23.16±0.01	23.71±0.11	23.87±0.03
	**T2**	23.20±0.01	23.64±0.017	23.73±0.12

Supplementary Table-2. Physicochemical Parameters assessed during the experiment.

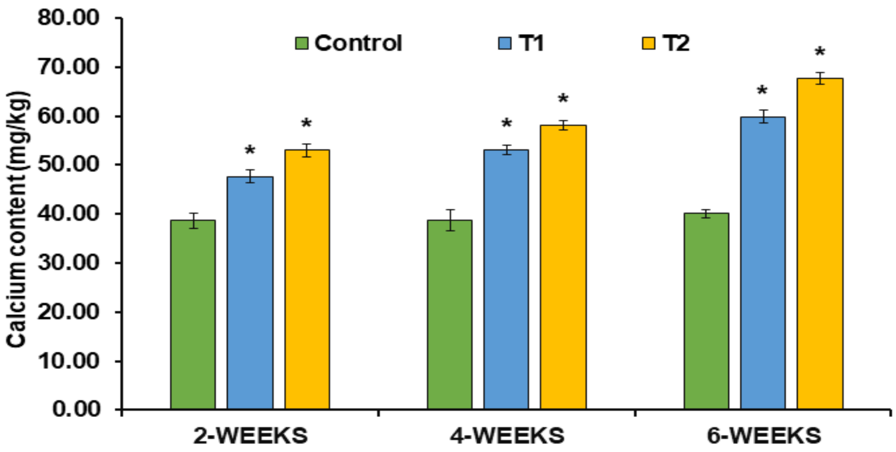

Supplementary Fig.1. Intracellular Calcium content in the liver tissues of *L. rohita* after 2, 4 and 6 weeks of exposure. (mean ±S.E.M., n=3 fish which were taken in triplicates); [*symbol represents the significant (p<0.05) difference from the control, analyzed using one-way ANOVA with Tukey's post hoc test].

a

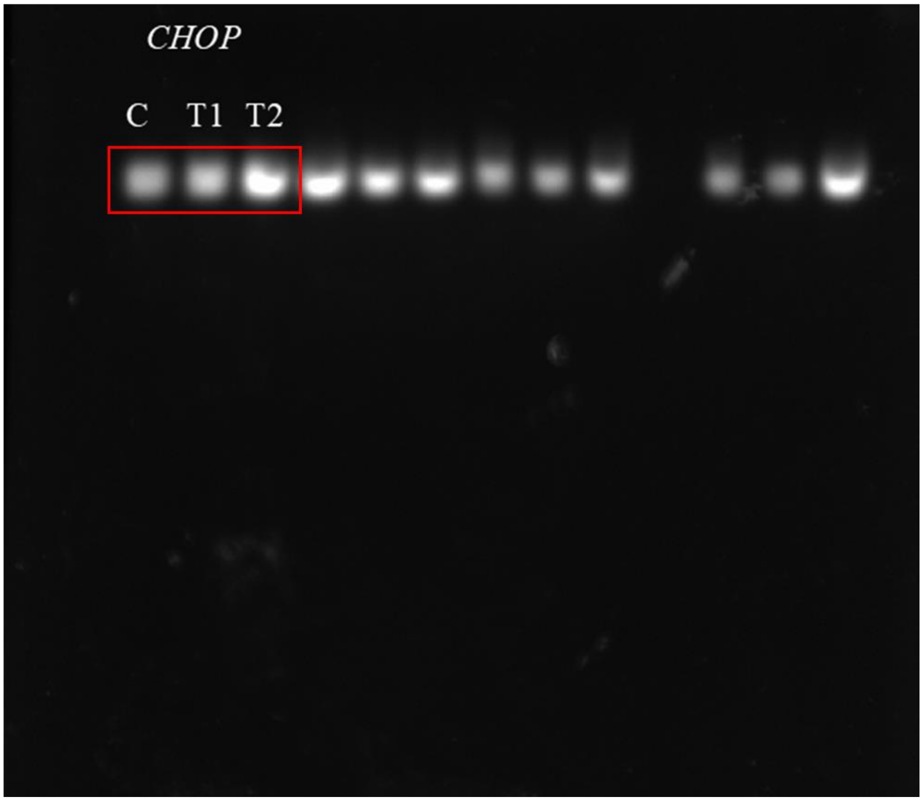

b

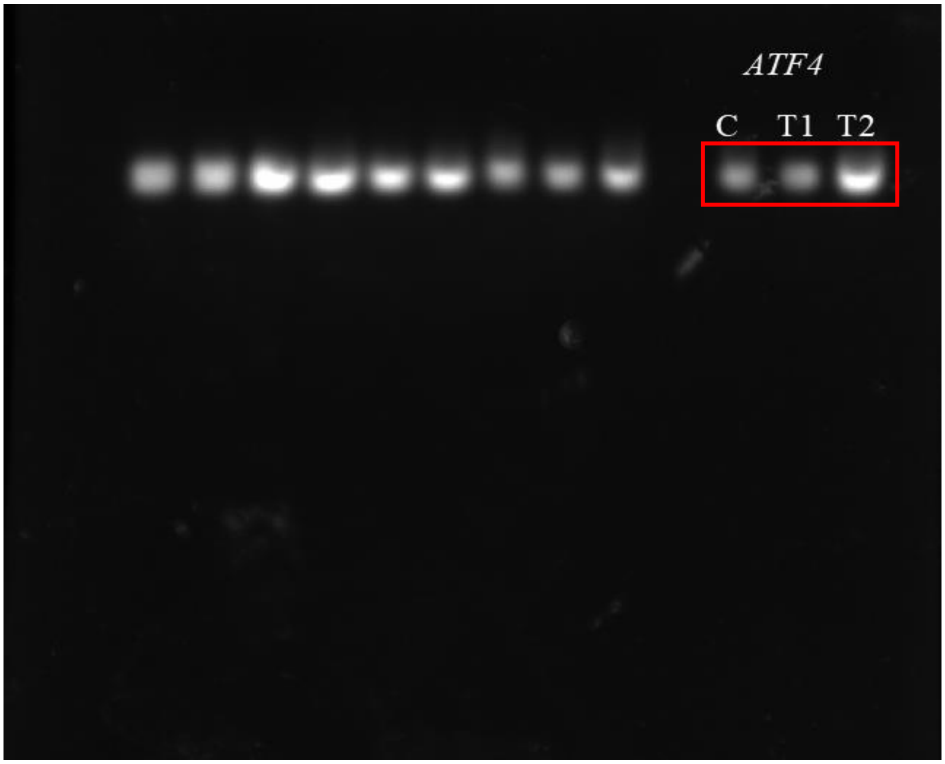

c

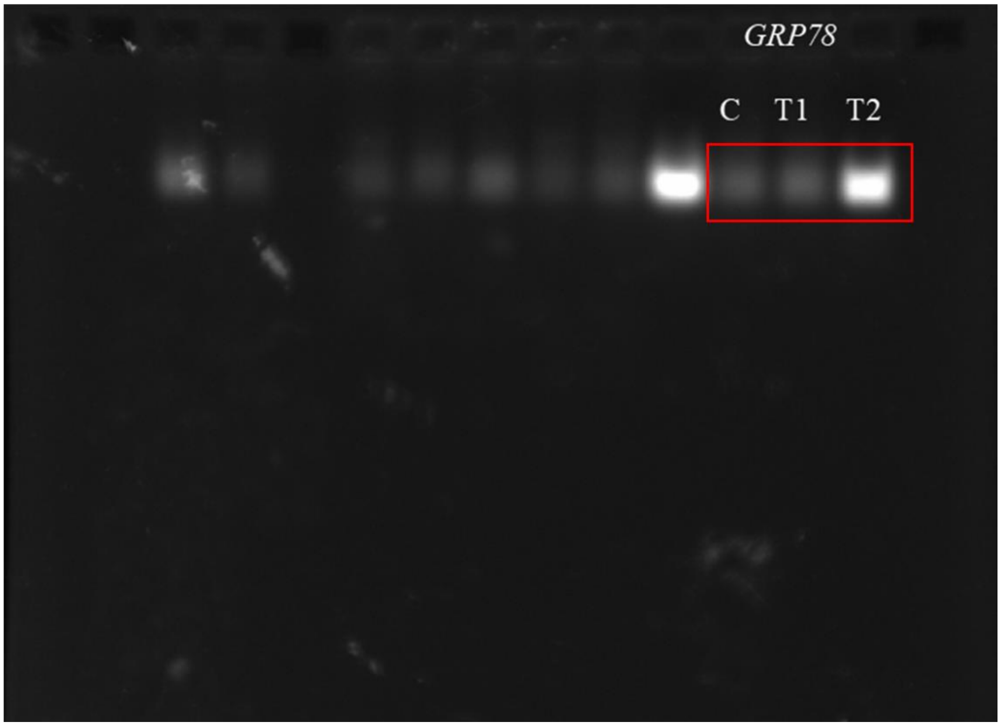

d

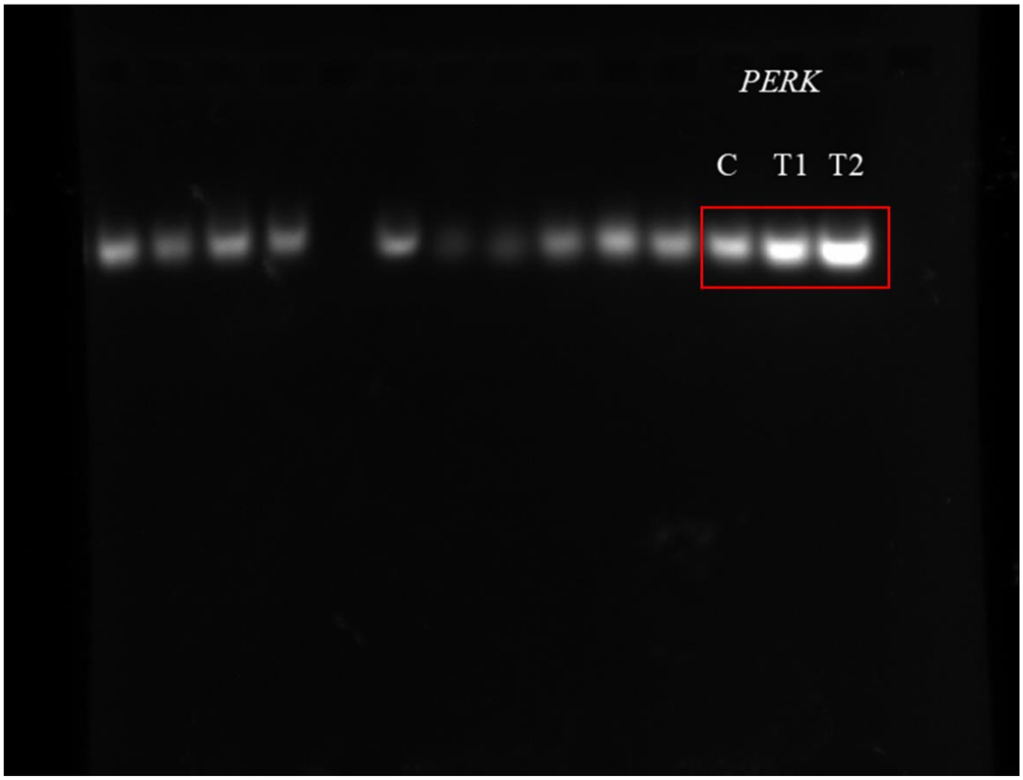

e

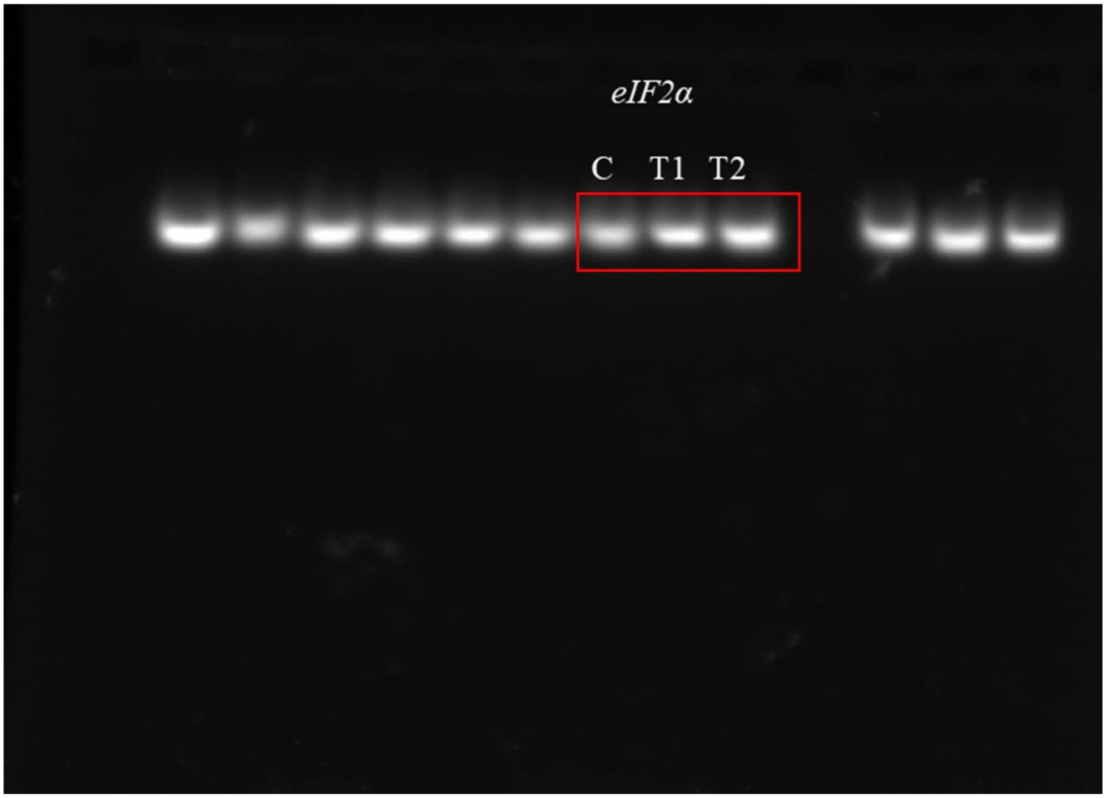

f

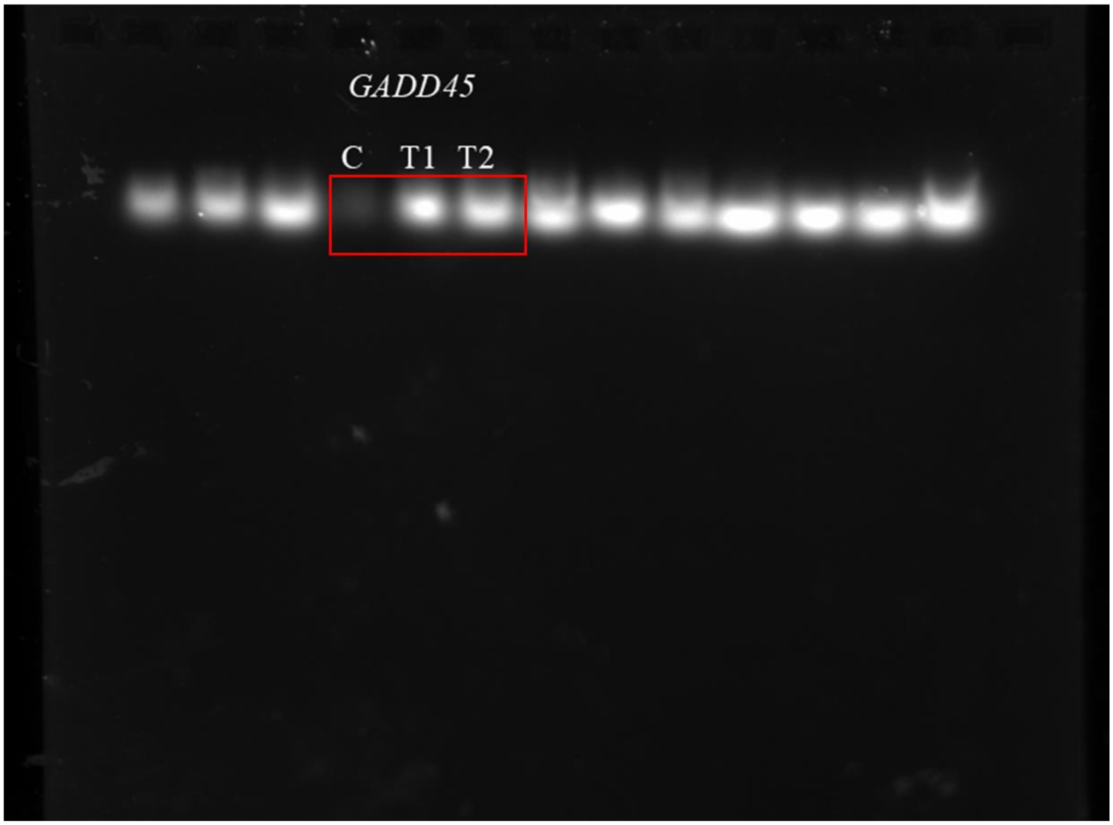

g

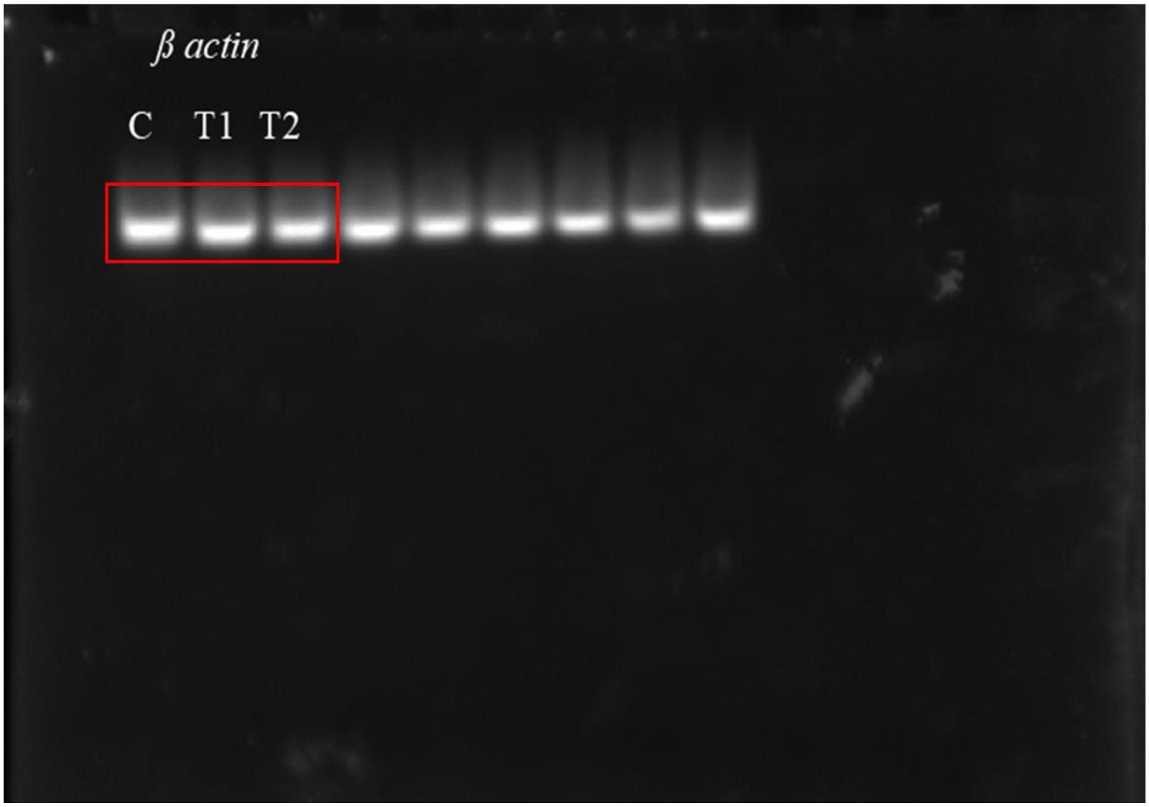

h

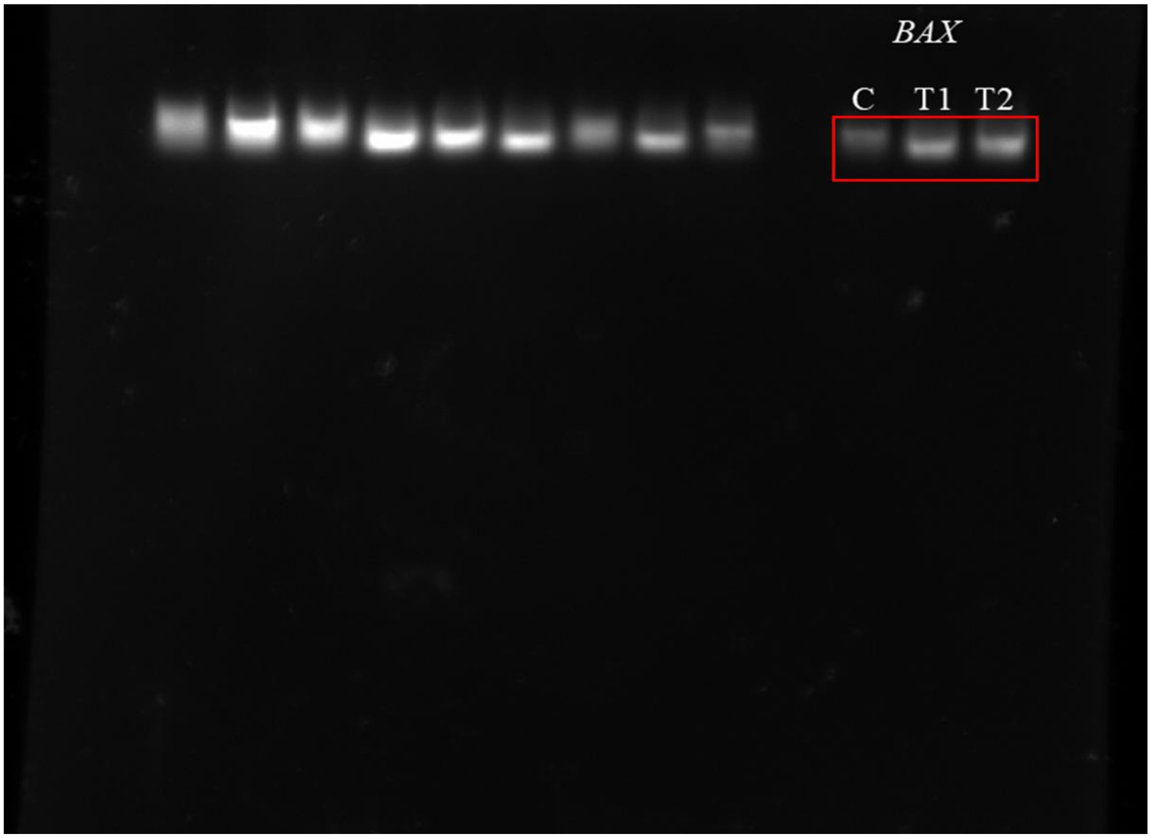

i

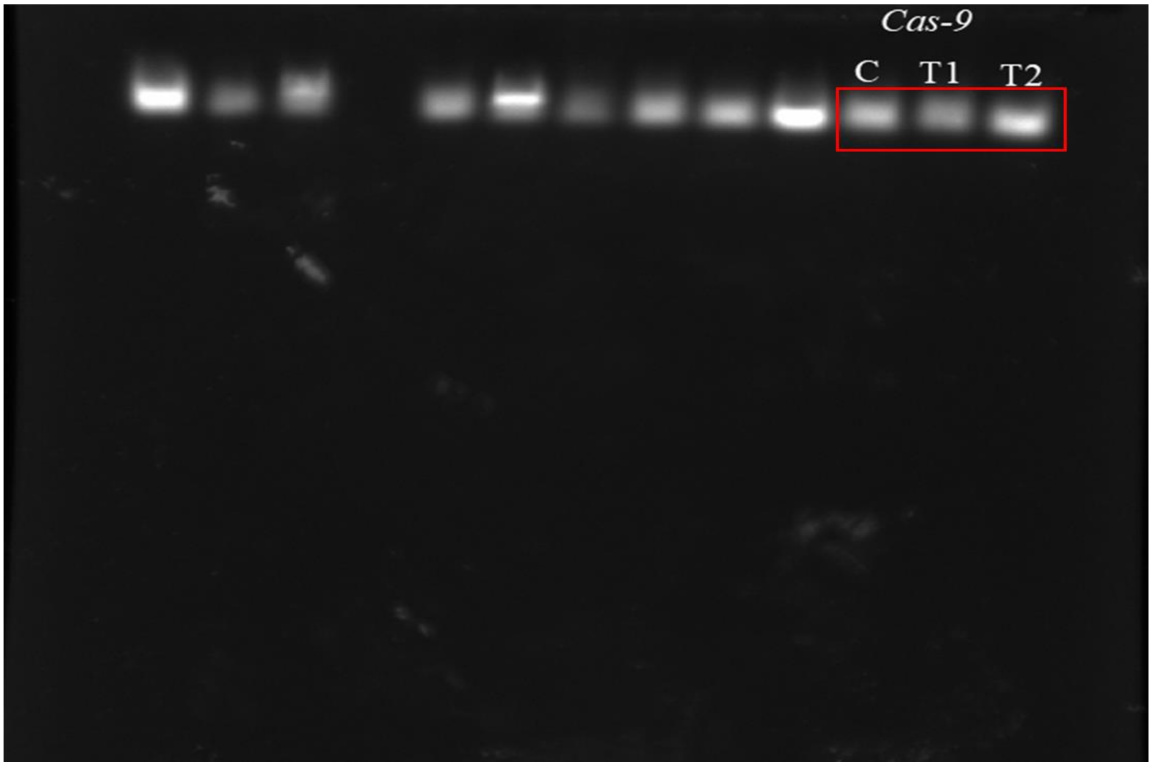

j

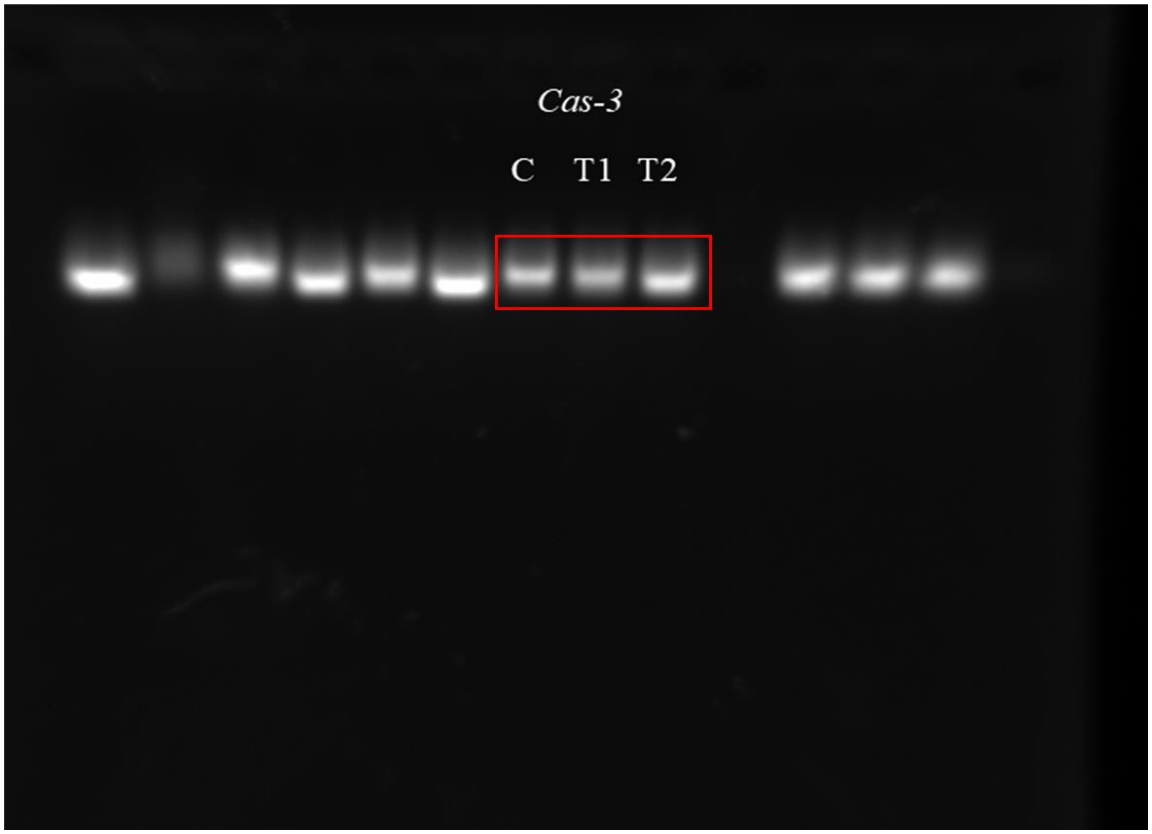

k

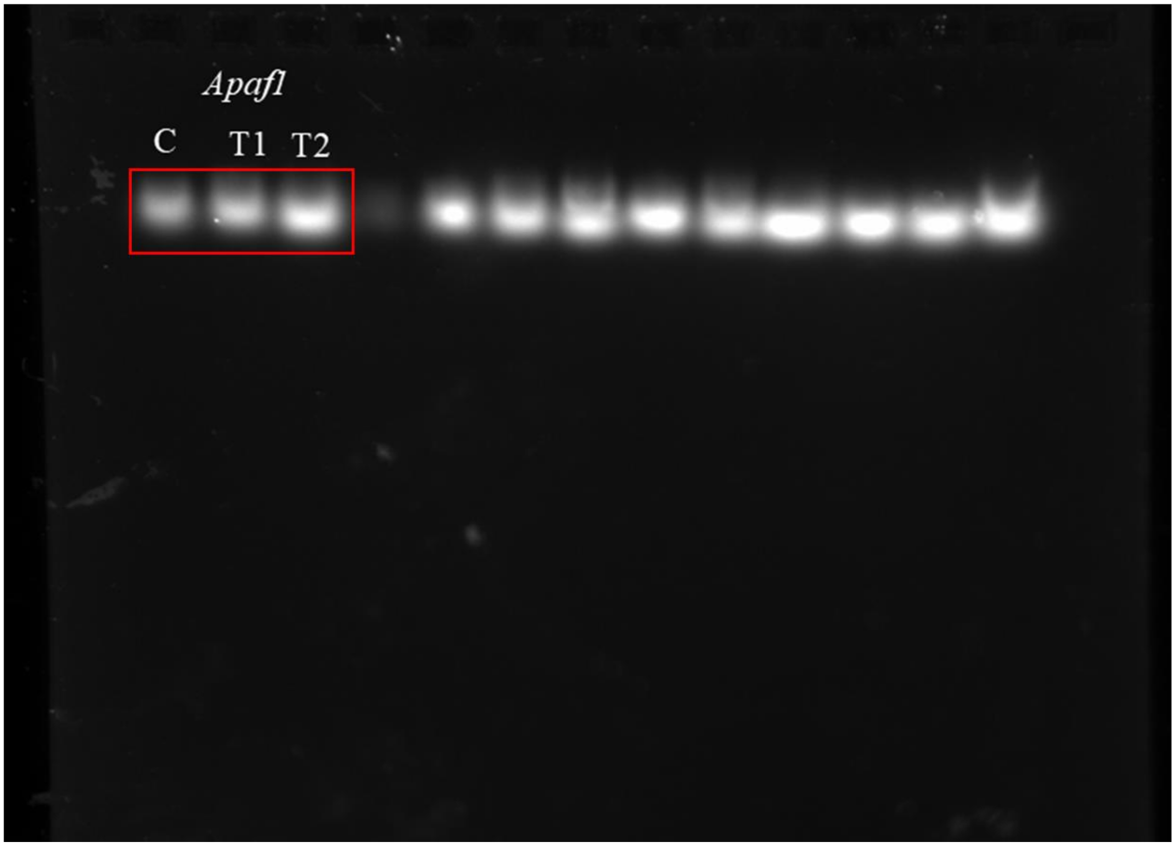

l

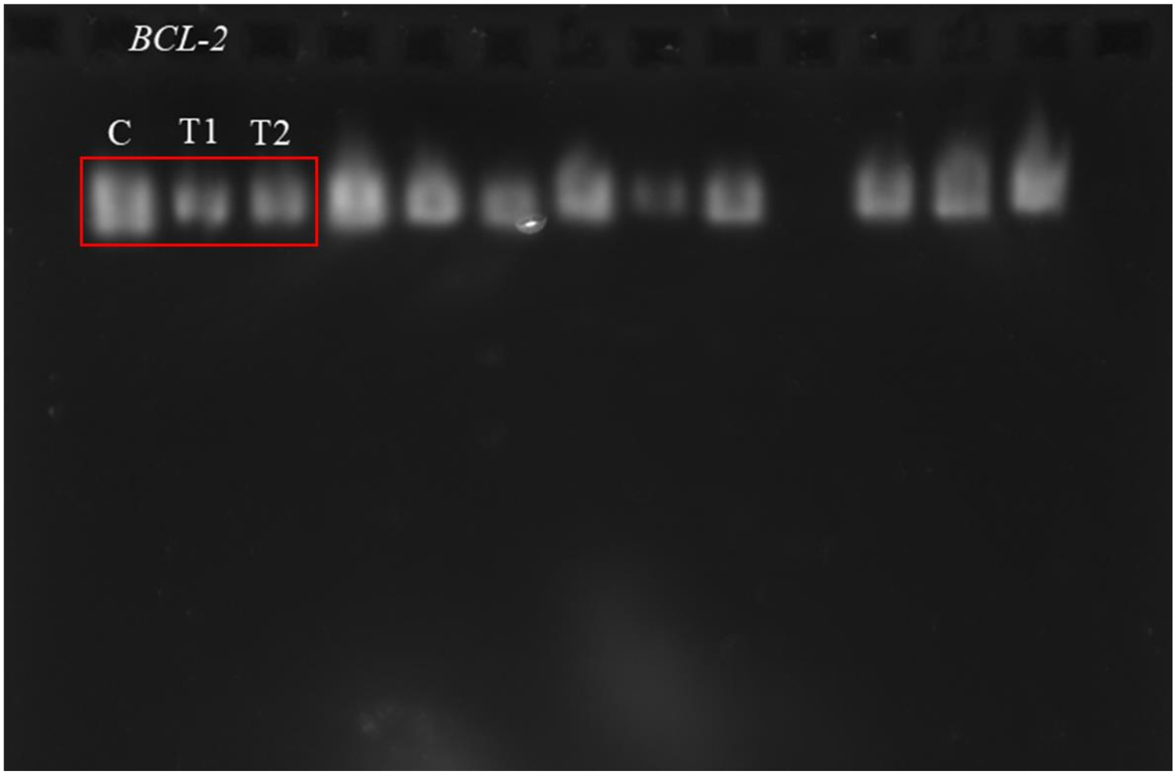

m

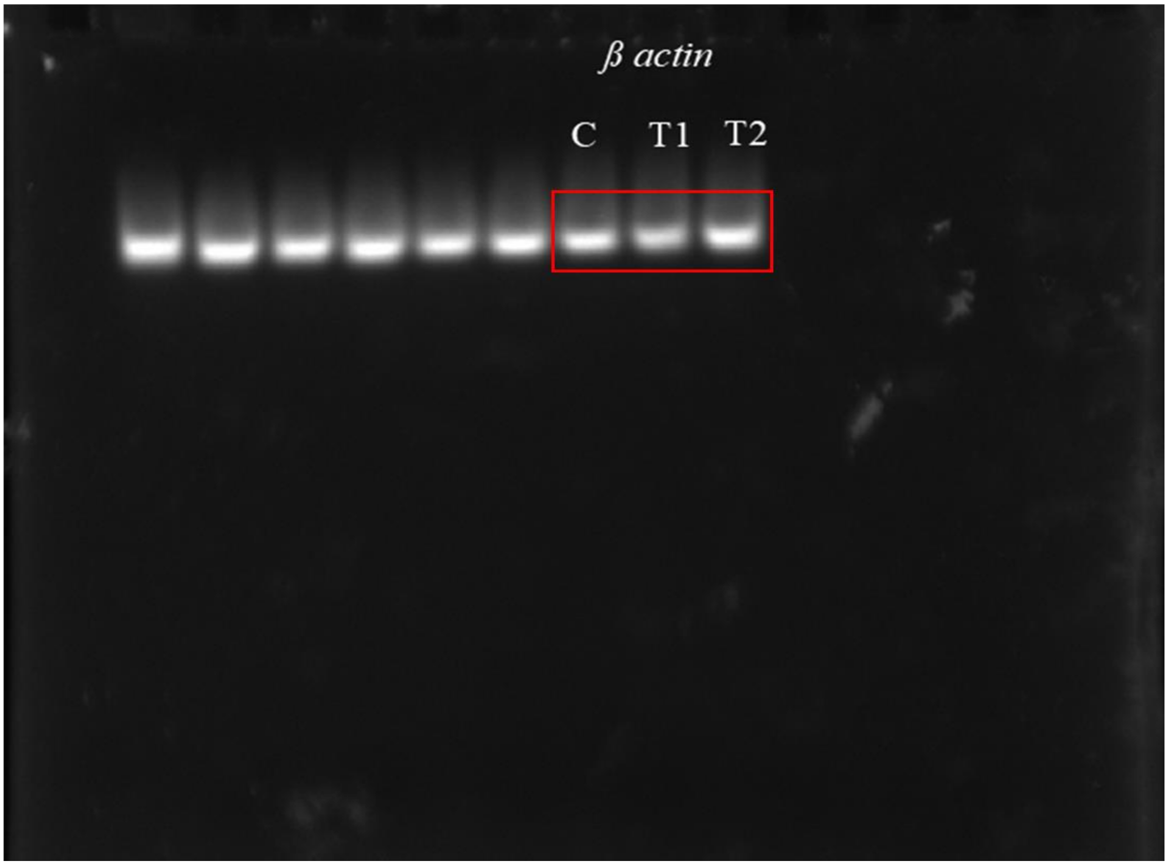

Supplementary Fig. 2. Full-length images of gel bands representing ER stress-related genes: (a) CHOP, (b) ATF4, (c) GRP78, (d) PERK, (e) eIF2α, (f) GADD45, and (g) β-actin. Apoptosis-related genes include (h) BAX, (i) CAS9, (j) CAS3, (k) APAF1, (l) BCL2, and (m) β-actin. Samples analyzed: Control, T1 (Treatment 1), and T2 (Treatment 2).
